# Age-specific and sex-specific risks for HCC in African-born persons with chronic hepatitis B without cirrhosis

**DOI:** 10.1097/HC9.0000000000000334

**Published:** 2023-12-01

**Authors:** Habiba Kamal, Michael Ingre, Per Stål, Gabriel Westman, Daniel Bruce, Heiner Wedemeyer, Ann-Sofi Duberg, Soo Aleman

**Affiliations:** 1 Department of Infectious Diseases, Karolinska University Hospital, Stockholm, Sweden; 2 Department of Medicine Huddinge, Karolinska Institute, Stockholm, Sweden; 3 Centre for Bioinformatics and Biostatistics, Karolinska Institute, Stockholm, Sweden; 4 Department of Upper GI Diseases, Karolinska University Hospital, Stockholm, Sweden; 5 Department of Medical Sciences, Section of Infectious diseases, Uppsala University, Uppsala Sweden; 6 SDS Life Science, Stockholm, Sweden; 7 Department of Gastroenterology and Hepatology, University of Hannover, Germany; 8 Department of Infectious Diseases, Örebro University Hospital, Örebro, Sweden

## Abstract

**Background:**

The international recommendations of HCC surveillance for African-born persons with chronic hepatitis B (CHB) without cirrhosis are divergent, probably due to scarce data on incidence rate (IR) for HCC.

**Methods:**

We assembled a cohort with prospectively collected data of Swedish residents of African origin with diagnosed CHB without cirrhosis at baseline from 1990 to 2015. Data from nationwide registers were used to calculate the sex-specific IR and IR ratio (incidence rate ratios) in relation to age, comorbidities, and birth region, using a generalized linear model with a log-link function and Poisson distribution.

**Results:**

Among 3865 African-born persons with CHB without cirrhosis at baseline, 31 (0.8%; 77.4% men) developed HCC during a median of 11.1 years of follow-up, with poor survival after HCC diagnosis. The mean age at HCC diagnosis was 46.8 (SD±14.7; range 23–79) in men. HCC IR exceeded the recommended surveillance threshold of 0.2%/year at ages 54 and 59 years in men and women, respectively, and at ages 20–40 years if HCV or HDV co-infection was present. African-born men with CHB had an incidence rate ratios of 10.6 (95% CI 4.4–31.5) for HCC compared to matched African-born peers without CHB, and an incidence rate ratios of 35.3 (95% CI 16.0–88.7) compared to a matched general population.

**Conclusions:**

African-born men with CHB without cirrhosis reached an IR of 0.2%/year between 50 and 60 years, and at younger ages if HCV or HDV co-infection was present. Our findings need further confirmation, and new cost-effectiveness analyses specific for young populations are needed, to provide personalized and cost-effective HCC surveillance.

## INTRODUCTION

HCC is the second leading cause of cancer-related mortality among men and the sixth among women in Africa, with overall 80% of HCC being attributed to HBV infection.^1^ It is estimated that 11 million African-born migrants reside in Europe, 5 million in Asia, and 3 million in Northern America.^2^ This has changed the epidemiology of chronic hepatitis B (CHB) in several low-endemic countries hosting African migrants.^3^ Diagnosis of HCC at an advanced stage has been previously reported in young men of African origin with CHB.^4,5^

Surveillance for HCC with biannual ultrasound +/− alfa-fetoprotein is currently recommended for persons at high risk for HCC to detect tumors at early and curable stages.^6^ Current guidelines have used a cost-effectiveness threshold incidence for the surveillance of 0.2% per year for patients with CHB without liver cirrhosis and 1.5% for those with cirrhosis, for recommendations on when to start surveillance for HCC.^7,8^ The knowledge about the incidence rates (IR) of HCC in African-born immigrants with CHB and without cirrhosis per age and sex is though scarce. Our research group in a previous study showed that the annual risk for HCC in men with CHB from Sub-Saharan Africa, including both those with cirrhosis and without cirrhosis, exceeded 0.2%/year from the age group of 50–59 years.^9^ The paucity of incidence rate data for HCC is a probable reason for discordant international recommendations regarding from which age to start HCC surveillance in persons of African origin with CHB and without cirrhosis, especially for men.^5^

The American Association for the Study of Liver Disease (AASLD) recommends HCC surveillance for African or African American men of age ≥ 40 years.^10^ In contrast, the Asian Pacific Association for the Study of the Liver (APASL) and the Canadian Association for the Study of the Liver (CASL) recommend surveillance of persons with CHB and African origin from 20 years of age, regardless of sex.^11,12^ The Chinese guideline recommends the surveillance of all persons with CHB, including persons without cirrhosis regardless of age.^13^ The European Association for the Study of the Liver (EASL) does not take into account any ethnicity, but recommends HCC surveillance based on the risk score of platelet-age-gender–HBV.^14^ This risk score has been validated in Caucasian and Asian populations, but not in an African population.^15,16^ The Swedish national guideline recommends HCC surveillance for CHB in men >40 years and women >50 years of African origin, especially if other risk factors are present.^17^

In this study, we included a nationwide cohort of 3865 African-born individuals with CHB without cirrhosis and investigated the IR of HCC in relation to age, sex, comorbidities, and region of birth. The risk in the study population was compared with the risk in individuals without CHB from the same country of origin, and from the general population, matched for age, sex, and county of residence.

## METHODS

### Study population

**FIGURE 1:**
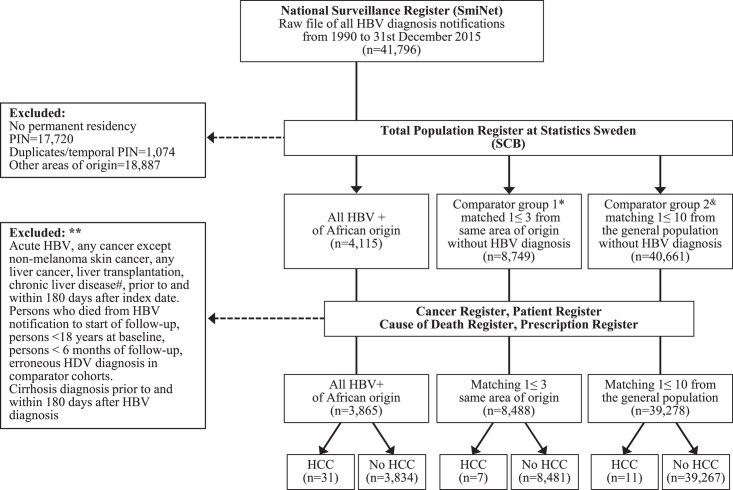
Flowchart of the study population. #chronic liver diseases included parasitic, hereditary, and autoimmune liver diseases; *Matched on age, sex, date of HBV notification, country, and area of origin. ^&^Matched on age, sex, date of HB notification, and county. **Participants were excluded in one step from the data set. Abbreviations: PIN, personal identification number; SCB, “Statistiska Centralbyrån” = Statistics Sweden.

A nationwide dataset including all persons who had been diagnosed with HBV infection through HBsAg positivity (n = 41,796) from January 1, 1990 to December 31, 2015 was constructed, using data from the National Surveillance Register SmiNet at the Public Health Agency (Flowchart in Figure 1). Only African-born persons with CHB and residency in Sweden at the date of diagnosis were included, then categorized by the country of birth into Northern, Eastern, Middle, Western, and Southern Africa, according to United Nations regional geographical definition.^18^ We included persons from the whole African continent, extended the follow-up time until the end of 2019 compared to our previous study,^9^ and excluded those with cirrhosis prior to or within 6 months after CHB diagnosis. Cirrhosis diagnosis was ascertained from the Patient Register using International Classification of Diseases (ICD) codes (ICD-9 and ICD-10) for cirrhosis at hospital discharge or outpatient specialty care, as described.^19^

### Linkage to national registers for outcomes

We linked the dataset of persons with CHB to validated national registries, the Patient Register, Cause of Death Register, Cancer Register, and Prescribed Drug Register at the National Board of Health and Welfare, described in our previous study.^9^ The PR contains prospectively updated national data of all hospitalizations including liver transplantations, discharge diagnoses (since 1964), and diagnoses from outpatient specialist care (since 2001). The diagnoses are recorded with ICD codes in well-validated registries. The Prescribed Drug Register has prospectively recorded all dispensed prescriptions, including antivirals against HBV infection, that is, nucleos(t)ide analogs (NA) or pegylated interferon, from Swedish pharmacies since 2005.

Persons with ≤ 6 months of follow-up, and persons who had at least 2 episodes (to rule out misclassification) with diagnoses of other liver diseases as hereditary hemochromatosis, autoimmune and parasitic causes, liver cancer, or any diagnoses of cancer except nonmelanoma skin cancer, and liver transplantation, prior to or within 6 months after CHB diagnosis were excluded (n = 219). The index date, that is, the start of follow-up at baseline, was set to the date of HBV notification to the Public Health Agency, plus a time window of 6 additional months, to avoid surveillance bias. The final cohort for the analysis constituted of 3865 persons with CHB.

Demographics and other parameters, including sex, age at HBV diagnosis, alcohol overconsumption, comorbidities such as diabetes mellitus, obesity, HDV, HCV, and HIV co-infections, as well as HBV treatment were collected from the mentioned registers through ICD and ATC (Anatomical Therapeutic Chemical) Classification system codes.

### Outcomes

The primary outcome was liver cancer (ICD codes as published in a prior work^19^) primarily retrieved from the Cancer Register, and the Death Register for combining data increases the coverage.^20^ Liver cancer is referred to as HCC in this study, as most liver cancers are HCC in a cohort with CHB.^20^ The study participants were followed until the first recorded date of HCC, death, liver transplantation, or December 31, 2019, whichever occurred first.

### Comparison cohorts

Two matched comparison cohorts without CHB diagnosis were obtained from Statistics Sweden. The matching date was the date of HBV notification and the comparators had to be alive and reside in Sweden at that date. First cohort: each subject with CHB was matched with up to 3 individuals (when possible) on birth year, sex, county of residence at notification, and country of origin (n = 8488). Second cohort: each subject with CHB was matched on birth year, sex, and county of residence in Sweden with up to 10 individuals from the Swedish general population (n = 39,267).

### Ethical permit

The Regional Ethical Review Board in Stockholm, Sweden, approved the study (Dnr 2015/1282-31). As this study is register-based using pseudonymized data, informed consent from the participants was waived.

## STATISTICAL ANALYSIS

Continuous variables were presented as mean (SD) when normally distributed, and as median [interquartile range (IQR)] when the distribution was skewed. Student-t test and Mann–Whitney test were used for the comparison of normal and skewed continuous variables, respectively. Categorical variables were presented as frequencies and proportions and were compared using the chi-Square test or Fisher exact test, whenever appropriate.

IR of HCC during follow-up were calculated as the number of new HCC diagnoses divided by the sum of person-years at risk and were reported as events per 100 person-years (PY) [eg, (number of new-onset HCC/person-years) *100] with 95% CI. To calculate IR and incidence rate ratios (IRR), a generalized linear model was applied to data, using a log-link function together with a Poisson error distribution and an offset with the logarithm of follow-up time constrained to a coefficient of 1. The model aimed to calculate the sex-specific age when IR would cross the currently recommended surveillance threshold of 0.2%/year. To that end, a base model was fitted with HCC as the dependent variable and independent variables indicating sex and age on a continuous scale (centered around the mean) together with the interaction age*sex. All terms in the model were significant with CIs that did not include unity (see results below). Sensitivity analyses indicated that the base model [Akaike Information Criterion (AIC) = 371] provided superior fit over a model with four discrete age groups (AIC = 384) and that adding polynomials (AIC = 373) or natural splines (AIC = 373) to the model to account for potential nonlinearity of age did not improve fit over the base model. The base model was used to calculate the sex-specific age when IR crossed above the surveillance threshold and was also used to test if adding comorbidities or regions of origin could add to the prediction.

All *p* values were two-tailed and statistical significance was set to *p*-value < 0.05. Analyses were performed using SAS (version 9.4, SAS Institute Inc., Cary, NC, USA), SPSS IBM Statistics (version 28.0), and R version 4.2.2.

## RESULTS

### Baseline characteristics

The baseline characteristics of the 3865 African-born persons with CHB and without liver cirrhosis at baseline are presented in Table 1. The mean (±SD) age at HBV diagnosis was 32.1 (± 11.2) years. Men constituted 58.6% of the cohort. The predominant area of origin was Eastern Africa (64.1%), followed by Western Africa (22.6%). Men had significantly more frequent HCV co-infection, alcohol overconsumption, diabetes mellitus, and drug misuse at baseline, and were more frequently prescribed anti-HBV therapies than women (all *p* = <0.05).

**TABLE 1:** Baseline characteristics of African-born persons with chronic hepatitis B (CHB) and without liver cirrhosis, living in Sweden

Characteristics	All (n, %)	Men (n, %)	Women (n, %)	*p*-value
Total	3865	2266 (58.6)	1599 (41.4)	< 0.001
Age at immigration, mean (SD), y^a^	27.5 (11.6)	27.5 (11.5)	27.6 (11.9)	0.89
Age at the start of follow-up, mean (SD), y	32.1 (11.2)	32.5 (11.2)	31.4 (11.2)	< 0.001
Age groups at the start of follow-up, y	—	—	—	< 0.001
18–29	1805 (46.7)	994 (43.9)	811 (50.7)	—
30–39	1252 (32.4)	715 (31.6)	537 (33.6)	—
40–49	531 (13.7)	398 (17.6)	133 (8.3)	—
> 50	277 (7.2)	159 (7.0)	118 (7.4)	—
HBV diagnosis date, y	—	—	—	< 0.001
1990–1999	861 (22.3)	469 (20.7)	392 (24.5)	—
2000–2009	1554 (40.2)	895 (39.5)	659 (41.2)	—
2010–2015	1450 (37.5)	902 (39.8)	548 (34.3)	—
Education level (years in school), y	—	—	—	< 0.001
≤ 9	1395 (36.1)	778 (34.3)	617 (38.6)	—
10–12	1246 (32.2)	756 (33.4)	490 (30.6)	—
> 13	858 (22.2)	567 (25.0)	291 (18.2)	—
Missing	366 (9.5)	165 (7.3)	201 (12.6)	—
African region of birth	—	—	—	0.90
Northern	261 (6.8)	152 (6.7)	109 (6.8)	—
Eastern	2478 (64.1)	1455 (64.2)	1023 (64.0)	—
Middle	240 (6.2)	152 (6.7)	94 (5.9)	—
Western	874 (22.6)	505 (22.3)	369 (23.1)	—
Southern	12 (0.3)	8 (0.4)	4 (0.3)	—
Comorbidities				
HCV co-infection	194 (5.0)	130 (5.7)	64 (4.0)	0.02
HDV co-infection	105 (2.7)	63 (2.8)	42 (2.6)	0.77
HIV co-infection	126 (3.3)	75 (3.3)	51 (3.2)	0.84
Alcohol overconsumption	93 (2.4)	75 (3.3)	18 (1.1)	< 0.001
Diabetes mellitus	326 (8.4)	221 (9.8)	105 (6.6)	< 0.001
Obesity	28 (0.7)	3 (0.1)	25 (1.6)	<0.001
Drug misuse	60 (1.6)	47 (2.1)	13 (0.8)	0.002
Co-medications				
Interferon therapy	39 (1.0)	32 (1.4)	7 (0.4)	0.003
Nucleos(t)ide analogs	260 (6.7)	178 (7.9)	82 (5.1)	<0.001
Statin	224 (5.8)	157 (6.9)	67 (4.2)	< 0.001
Aspirin	135 (3.5)	81 (3.6)	54 (3.4)	0.74
Follow-up time, y				
Mean (SD)	12.4 (6.7)	12.0 (6.6)	13.1 (6.8)	< 0.0001

Note: The characteristics for all and grouped by sex are shown, with a *p*-value for comparison between men and women.

a The age or year at first immigration event, if >one event of immigration.

Abbreviations: CHB, chronic HBV; DM, diabetes mellitus.

The characteristics of persons with CHB by African region of birth are presented in Supplemental Table S1, http://links.lww.com/HC9/A683. Middle, North, and South African persons had significantly more frequent HCV co-infections, compared to their peers from Eastern and Western Africa (*p* = <0.001). Characteristics of the 2 comparator groups (men) are presented in Supplemental Table S2, http://links.lww.com/HC9/A683.

### Characteristics of persons who developed HCC

**FIGURE 2:**
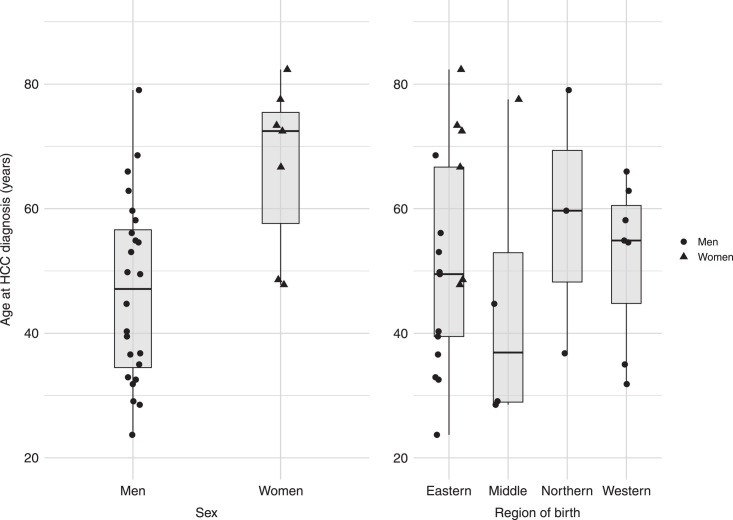
Box plot showing age at HCC diagnosis in African-born persons with chronic hepatitis B (CHB) and without cirrhosis, by sex and region of birth. Dots and triangles represent individual persons with developed HCC (n = 31), and the middle line (thickened) in the box plot represents the median age at HCC diagnosis. The upper and lower edges of the box represent the 25th and the 75th percentile, respectively, and the upper and lower error bars represent the minimum and maximum age, respectively. The y-axis represents age in years at HCC diagnosis. The x-axis represents the region of birth and sex. CHB, chronic hepatitis B.

Thirty-one (0.8%) persons developed HCC during a median follow-up (IQR) of 11.1 (6.6–17.6) years, corresponding to 48,066 person-years. The majority were men (n = 24, 77.4%) (Supplemental Table S3, http://links.lww.com/HC9/A683). Seventy-four persons (1.9%) were diagnosed with cirrhosis during follow-up. The mean±SD age at HCC diagnosis was 51.4±16.6 y, with a significantly lower age in men (mean 46.8±14.7; range 23–79 y) than in women (mean 67±13.7; range 48–82 y) (*p* = 0.03) (Figure 2). The mean (±SD) age at immigration for persons who developed HCC was significantly higher at 35.5 (± 16.4) years, compared to 27.5 (± 11.6) years for those who did not develop HCC (*p* = 0.01). For men, the difference was though not statistically significant when comparing the age of immigration in those with and without HCC (30.4 vs. 27.5 y of age, *p* = 0.22).

Fifteen persons with HCC (48%) had received cirrhosis diagnosis prior to HCC diagnosis. However, 9 (60%) of them had received cirrhosis diagnosis ≤ 6 months prior to HCC diagnosis, including 8 (8/15, 53%) with ≤ 3 months. Fifteen persons (48%) received NA prior to HCC diagnosis for a median duration of 11.0 months (IQR, 6.7–15.3).

Development of HCC < 40 years of age was seen in 10 men (10/24, 42%). Among them, all 5 (50%) with liver cirrhosis at the time of HCC had received the cirrhosis diagnosis ≤ 3 months prior to HCC diagnosis. The characteristics of persons who developed HCC, and subgrouped by area of origin, are shown in Supplemental Table S4, http://links.lww.com/HC9/A683.

### Risk for development of HCC

**FIGURE 3:**
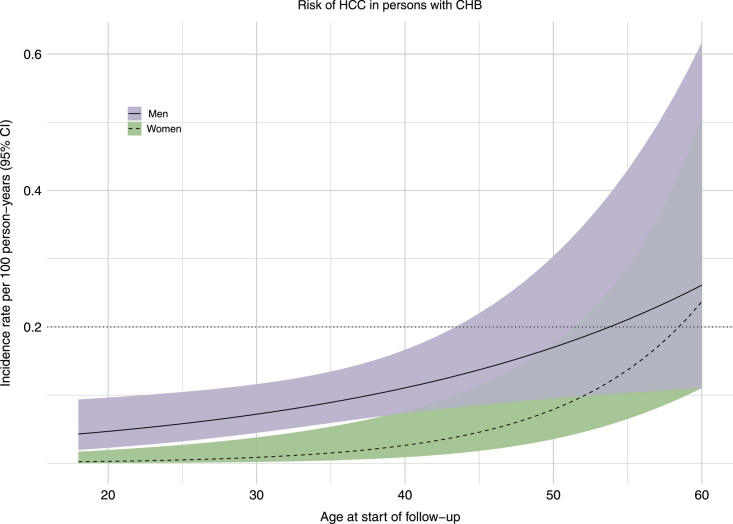
The incidence rate of HCC per 100 person-year in African-born persons with CHB and without cirrhosis, by sex. The x-axis represents the age at baseline on continuous scale. The y-axis represents the incidence rate of HCC per 100 persons per year. The dotted horizontal line marks an incidence rate of 0.2% of cost-effective HCC surveillance in individuals without cirrhosis. The lilac and green zones represent the 95% CI for the mean incidence rate in men and women, respectively. The 0.2% IR was exceeded in men at age 54 years (IR = 0.20/100PYs, 95% CI: 0.10–0.40) and in women at age 59 years (IR = 0.21/100 PYs, 95% CI: 0.10–0.45). Age*sex interaction was IRR (0.94, 95% CI: 0.88–0.99) suggesting that the increased risk was more pronounced in younger age in men and attenuated with older age compared to women. Abbreviations: CHB, chronic hepatitis B.

To estimate the age-specific and sex-specific risk of HCC a base model was fitted, showing a significant IRR for men (IRR = 7.13; 95% CI: 2.08–42.31), age in years (IRR = 1.12, 95% CI: 1.07–1.18) and the interaction age* sex (IRR = 0.94; 95% CI: 0.88–0.99). This indicates that men were at higher risk for HCC than women, and that the risk increases with age but that this increase was attenuated with older age in men (Table 2). Figure 3 shows the predicted IR, illustrating also that the increase of risk in men was most pronounced at younger ages.

**TABLE 2:** Incidence rate ratios for HCC derived from Poisson regression models presenting the association of variables in a chronic hepatitis B cohort of persons of African origin

	Univariable		Base model (multivariable)		Adjusted estimates^a^	
Predictors	IRR (95% CI)	*p*	IRR (95% CI)	*p*	IRR (95% CI)	*p*
Base Model						
Age (centered)	1.07 (1.04–1.09)	0^b^	1.12 (1.07–1.18)	0^b^	– (–)	–
Men	2.66 (1.21–6.68)	0.02^b^	7.13 (2.08–42.31)	0.01^b^	– (–)	–
Age^b^ men	– (–	–	0.94 (0.88–0.99)	0.03^b^	– (–)	–
Comorbidities						
HCV co-infection	4 (1.49–9.14)	0.002^b^	– (–)	–	**2.75** (**1.01**–**6.37**)	**0.03** ^b^
HDV co-infection	4.27 (1.02–12)	0.02^b^	– (–)	–	**4.47** (**1.06**–**12.84**)	**0.02** ^b^
HIV co-infection	1.99 (0.32–6.6)	0.3	– (–)	–	2.01 (0.32–6.73)	0.3
DM	1.81 (0.61–4.33)	0.2	– (–)	–	0.97 (0.32–2.43	>0.9
African region of origin (vs. Eastern)						
Middle	2.73 (0.79–7.39)	0.07^b^	– (–)	–	**3.82** (**1.08**–**10.65**)	**0.02** ^b^
Northern	1.6 (0.37–4.76)	0.5	– (–)	–	1.73 (0.4–5.3)	0.4
Western	1.15 (0.44–2.66)	0.8	– (–)	–	1.77 (0.66–4.32)	0.2

a Adjusted estimates are univariable estimates adjusted for the Base Model.

b Statistically significant estimates in the adjusted model are presented in bold.

Abbreviation: DM, diabetes mellitus; IRR, incidence rate ratios; *p*, *p*-value.

Overall, men had an IR of 0.09/100PYs (95% CI: 0.06–0.13), while women had an IR of 0.03/100PYs (95% CI: 0.01–0.07). Men exceeded the currently recommended threshold for HCC surveillance (0.2%/y) at 54 years of age (IR = 0.20/100PYs, 95% CI: 0.10–0.40), while women did so at 59 years of age (IR = 0.21/100PYs, 95% CI: 0.10–0.45) (Figure 3).

Table 2 shows the HCC risk estimates of comorbidities with HCV, HDV or HIV co-infection and diabetes, adjusted for the predictors of the base model (age, sex, and age*sex) indicating increased risk for co-infection with HCV (IRR = 2.75, 95% CI: 1.01–6.37) and HDV (IRR = 4.47; 95% CI: 1.06–12.84), but not for HIV (IRR = 2.0, 95% CI: 0.32–6.73) or diabetes (IRR = 0.97, 95% CI: 0.32–2.43). Supplemental Figure S1, http://links.lww.com/HC9/A684, indicates that the increased risk with HCV or HDV co-infection placed men with these co-infections above the recommended surveillance threshold at the age of 33 years (IR = 0.20/100PYs, 95% CI: 0.08–0.48) and 20 years (IR = 0.21/100PYs, 95% CI: 0.05–0.75), respectively. Women reached the threshold at 51 and 46 years of age, respectively.

A similar analysis of the region of origin (Table 2) showed that persons from Middle Africa have an increased risk of HCC (IRR = 3.82, 95% CI: 1.08–10.65) compared to the Eastern region. The risk was similar when comparing those from Northern (IRR = 1.73, 95% CI: 0.40–5.30) or Western regions (IRR = 1.77, 95% CI: 0.66–4.32) to Eastern Africa. Predictions of HCC per region can be found in Supplemental Figure S1b, http://links.lww.com/HC9/A684.

The incidence rate of HCC per sex for persons with CHB and comparators are shown in Supplemental Figure S2, http://links.lww.com/HC9/A684.

### Survival of persons with CHB who developed HCC

Twenty-three (74%) African-born persons with CHB and HCC died during follow-up. Diagnosis of HCC was recorded on the same date as for death in 42% (13/31, 10 men and 3 women), with the retrieval of data from the Death Register. For the other, the median (IQR) survival time after HCC diagnosis was 8.2 (2.9–89.2) months, with similar survival time in men compared to women (Supplemental Table S4, http://links.lww.com/HC9/A683). The median (IQR) survival time for men < 40 years at HCC diagnosis was 4.4 (1.6–39.9) months, which was numerically lower than for men > 40 years with 25.6 months (*p* > 0–05). One man (4.2%) and one woman (14.3%) with an HCC diagnosis received a liver transplant, respectively.

### Risks of HCC compared to comparators

African-born men with CHB had 10.6 times the risk of developing HCC compared to matched African-born peers without CHB (IRR = 10.6, 95% CI: 4.4–31.5) and 35.3 times the risk of developing HCC compared to the matched general population (IRR = 35.3, 95% CI: 16.0–88.7). For African-born men with age < 40 years, the corresponding figures were 30.3 times the risk compared to African-born peers without CHB (IRR = 30.3, 95% CI: 4.0–230.5) and 141 times the risk of developing HCC compared to the matched general population (IRR = 141, 95% CI: 28.4–2559). African-born women with CHB demonstrated 7.4 times the risk compared to matched African-born peers without CHB (IRR = 7.4, 95% CI: 1.8–49.8) and 17.7 times the risk compared to matched women from the general population (IRR = 17.7, 95% CI: 5.3–67.4). Results are not shown in the tables.

## DISCUSSION

This study of 3865 African-born individuals with CHB and no cirrhosis at baseline using prospectively collected nationwide data showed that the IR of HCC exceeds the currently used threshold of 0.2% per year at 54 years in men and 59 years in women, using our statistical model. The cutoff age for which IR exceeds 0.2% per year was though younger if HCV or HDV co-infection was present with IR exceeding 0.2% at the age group of 20–30 years in men.

The decision from which age to start HCC surveillance in African-born persons, especially for men, is a clinical challenge, with diverging age-specific and sex-specific international recommendations.^10,14^ There is a paucity in the literature about the IR data of HCC per age and sex in African-born individuals with noncirrhotic CHB, in contrast to Asian or Caucasian populations, which could have contributed to the discordant guidelines.^21^ Moreover, few longitudinal studies have investigated the incident risk of HCC in African immigrants with CHB.^22^ Our study with estimates of sex-specific and age-specific IR can therefore provide some evidence for making clinical recommendations for HCC surveillance in this population. Given the occurring development of HCC in young men (even if low IR) with poor survival at late HCC diagnosis, in persons who would otherwise have gained many years of life expectancy if early HCC diagnosis with curative treatment, it is questionable whether the currently used cost-effective threshold of IR 0.2% is appropriate for this group. To our knowledge, there are no published data describing the cost-effectiveness analyses behind this threshold, despite current usage for recommendations in international guidelines.^10^ We have, in this study, described the age-specific and sex-specific IR, but it is nevertheless difficult to draw any firm conclusion from our model about the optimal age to start HCC surveillance in African men with CHB and no cirrhosis. The results need to be confirmed in further studies, and new cost-effectiveness analyses are probably needed for updated recommendations of HCC surveillance in African-born men.

Several studies in and outside the African continent pointed to the young age and advanced disease stage at HCC diagnosis in the population of African descent, regardless of CHB diagnosis.^5^ In a US-based study, African Americans tended to be younger, with more frequent HIV co-infections, and at an advanced stage compared to Caucasians at HCC diagnosis.^23^ In a large US Veteran’s Administration cohort study, patients with CHB with HCC and no cirrhosis were more likely to be non-White (African American or Asian), had a family history of HCC and hypertension compared to those with cirrhosis and HCC.^24^ US-born African Americans with CHB tended to be older age, have a more frequent history of sexually transmitted diseases, and illicit use of drugs and tattoos than foreign-born African Americans with CHB.^25^ It is plausible that this young age at HCC diagnosis reflects partially the younger age distribution in the migrated African-born population. Genetic susceptibility, early HBV infection, other co-infections, and environmental factors such as aflatoxin B1 have been associated with HCC in the African population.^26,27^ In this study, there was a significant difference in the age at immigration to Sweden (as an indicator for exposure time in Africa) in persons with or without HCC in the whole population, but no difference was seen in men with or without HCC. According to a forecasting analysis, Hispanics and African Americans are predicted to have the highest HCC incidence rates, especially in the age group of 35–49 years among African Americans by 2030 in California.^28^ The low incidence of cirrhosis or HCC in our cohort is in line with the results from a younger West African cohort with CHB and a recent register-based Danish study of persons with CHB of any origin, while in contrast to higher rates present in US studies including African American or Black population with CHB.^29–31^ The low incidences in our cohort could possibly be due to the young population, the healthy migrant effect, free health care in Sweden with good coverage of antiviral treatment to prevent the progress of liver fibrosis in the CHB population, or the underestimation of these events using ICD codes in the registers. Also, unrecognized cirrhosis may be present in our cohort, as a recent Swedish analysis has shown that such is frequent at the time of HCC diagnosis in persons with viral hepatitis.^3^

In Europe, African migrants, especially from Sub-Saharan Africa, harbor the highest prevalence of CHB.^3^ The African population constitutes 15%–45% of the published European cohorts of persons with CHB,^9,32,33^ but the association of African origin with HCC has not been explored.^34,35^ The poor survival in men with HCC in our study indicates a rather late diagnosis of HCC at an advanced stage, which is in line with the description of advanced disease stage at HCC diagnosis in Africans, especially for men from Sub-Saharan Africa.^23,36^ Men had more prevalent comorbidities than women in our study. Diabetes mellitus was not associated with an increased risk of HCC in our cohort in contrast to the finding of other studies,^37^ which might be due to the few HCC outcomes limiting the power to detect such an association. Co-infections with HBV/HCV, HIV, and HDV have been shown to have a synergistic effect on HCC risk and are historically more prevalent in these populations with possible late linkage to care, suboptimal treatment uptake, and adherence.^38^ It is unclear if there was any difference between men and women regarding performed HCC surveillance in our cohort, or if certain subgroups had a higher risk of missed cirrhosis diagnosis. In a retrospective analysis of patients with heterogenous HCC etiologies, African Americans had 2 times higher odds of unrecognized cirrhosis, and less likelihood to undergo HCC surveillance compared to Caucasian peers, agreeing with findings from a contemporary French case series.^24,39^ In the latter study, where most participants (60%) were from Africa, 14% of HBV-related HCC developed in noncirrhotic liver, with significantly lower age among patients without cirrhosis compared to those with cirrhosis (51 vs. 58 y, respectively).^39^

In our study, 51% of the persons with HCC did not have cirrhosis diagnosis at the time of HCC diagnosis. Owing to the oncogenic potential of HBV, ~20–30% of HCC can develop in noncirrhotic livers.^21^ Forty-nine percent of the persons with HCC had received cirrhosis diagnosis prior to HCC diagnosis. However, in 60% of these, the cirrhosis diagnosis was received rather late, ≤ 6 months prior to HCC diagnosis, possibly reflecting the difficulty to diagnose cirrhosis timely, especially in young men. Poor survival after HCC diagnosis with a median survival of 8.2 months may indicate that these men have not been subject to HCC surveillance prior to HCC diagnoses, with HCC being diagnosed at late stages. The late diagnosis of HCC reflects probably the difficulty to accurately predict the HCC risk in this young African population with no cirrhosis using risk-based HCC surveillance, nevertheless the current Swedish guidelines recommend surveillance from age 40 in men. Despite the free health care in Sweden, we cannot though rule out any disparity in health care due to language barriers or limited socioeconomics, which has been shown in other studies with correlation to poor survival after HCC diagnosis.^23^

Our findings highlight the importance of early identification of persons at increased risk of fibrosis progression for timely treatment.

In our model, we demonstrated an incidence rate > 0.2%/year at younger ages when HCV or HDV co-infection was present. The HCC cases were however few, resulting in a wide CI for the effect size. The elevated HCC IR in those with co-infections in our study is in line with prior estimates of persons with CHB of diverse origins and thus not only in those of African origin.^35^

Our study has some limitations. The few outcomes (only 31 cases of HCC) have limited the statistical power of our analyses, especially on a subgroup level. This despite being a rather large cohort, consisting of 3865 African-born persons without cirrhosis at baseline, with a long follow-up time of a median of 11.1 years. The low HCC incidence rate among persons with CHB without cirrhosis, leading to few HCC outcomes has been also apparent in other large cohorts.^24^ The number of persons with co-infections was few in this cohort, which limits the study to draw any firm conclusion about the effect of co-infections on the risk of HCC. The subgroups from the different African regions were not evenly distributed, with the majority from Eastern Africa and a limited sample size from some other regions. More studies are needed to study the risk in people from different parts of Africa. We cannot rule out the remaining unaccounted confounders. Related to the nature of this population-based register analysis, we did not assess the individual viral and host risk factors that might affect HCC development. Nevertheless, the effect of NA on the risk of HCC was not assessed. Individuals with more advanced liver disease, who developed cirrhosis, with a family history of liver cancer and other risk factors are prescribed NA and an unmatched analysis not considering these confounders might produce biased estimates. However, the international recommendations include only age, sex, and ethnicity as decision parameters to start HCC surveillance in clinical practice. Thus, we could in the context of these parameters explore the relevance of cutoff ages mentioned in the current guidelines of HCC surveillance. It is plausible that we might have underestimated the prevalence of cirrhosis at the index date by the usage of ICD codes for cirrhosis diagnosis, thus overestimating the risk of HCC in our cohort of patients with noncirrhotic CHB. Even with this potential overestimation, the IR for HCC exceeds 0.2%/year for men at > 50 years of age, with a substantially higher cutoff age than the recommended starting age for HCC surveillance in 20 or 40 years for African men in some international guidelines.^11,12^ We have assumed the cost-effective threshold IR to be 0.2%/year for persons with noncirrhotic CHB, as used for HCC surveillance in AASLD or EASL guidelines.^10,14^ We acknowledge, though, the need for contemporary cost-effectiveness studies and better risk stratification to support this cutoff data rather than a “one-size-fits-all” approach.^40^

The strengths of our study include the nationwide population-based design and usage of prospectively collected data, with the limitation of selection bias. Also, the abovementioned large sample size and long follow-up time allowed estimation of age-specific and sex-specific IR in our model, in contrast to previously published studies of patients with CHB with different origins or smaller cohort studies.^41^ While some previous studies have consisted mostly of men, we have data of both sexes with generalizability to both populations.^5,24^ Our findings may be generalized to other low-endemic settings, although noting the heterogeneity of African populations living with CHB after migration to the West. To limit surveillance bias, we started the follow-up 6 months after the index date and excluded all deaths, liver cancer, and liver transplantation.

In conclusion, our model indicates that the annual HCC risk of 0.2% is exceeded between age 50 and 60 years in African-born individuals with noncirrhotic CHB in Sweden, and with an earlier age if co-infection with HCV or HDV is present. The risk for HCC was not negligible in young men, with poor survival in those who developed HCC. There is however a need to further explore these risks and risk factors for HCC in young African men with CHB to be able to recommend the starting age for HCC surveillance in this population. There is also a need for new cost-effectiveness analyses specific to this young population at risk for HCC to be able to provide personalized and cost-effective surveillance.

## Supplementary Material

SUPPLEMENTARY MATERIAL
