# Supplementary material for: Age-specific and sex-specific risks for HCC in African-born persons with chronic hepatitis B without cirrhosis
Source: Hepatol Commun. 2023 Dec 1;7(12):e0334. doi: 10.1097/HC9.0000000000000334 (PMC10697596; doi:10.1097/HC9.0000000000000334)
Supplement: SUPPLEMENTARY MATERIAL [file hc9-7-e0334-s001.docx]

**HEP4-23-0604**

**Supplementary Tables**

- Table S1: Baseline characteristics of African-born individuals with CHB without liver cirrhosis per African region of birth.
- Table S2. Baseline characteristics of African-born men with CHB, and in comparator cohorts from the same area of origin and the general population without HBV infection.
- Table S3: Characteristics and survival of African-born persons with CHB who developed hepatocellular carcinoma, by sex.
- Table S4: Characteristics and survival of persons with CHB who developed HCC per African region of birth.

| ***Table 1.* *Baseline characteristics of African-born persons with chronic hepatitis B (CHB) and without liver cirrhosis, living in Sweden. The characteristics for all and grouped by sex are shown, with P-value for comparison between men and women.*** | | | | |
| --- | --- | --- | --- | --- |
| **Characteristics** | **All (n, %)** | **Men (n, %)** | **Women (n, %)** | **P-value** |
| **Total** | 3865 | 2266 (58.6) | 1599 (41.4) | <0.001 |
| **Age at immigration, mean (SD), years^¶^** | 27.5 (11.6) | 27.5 (11.5) | 27.6 (11.9) | 0.89 |
| **Age at start of follow-up, mean (SD), years** | 32.1 (11.2) | 32.5 (11.2) | 31.4 (11.2) | <0.001 |
| **Age groups at start of follow-up, years** |  |  |  | <0.001 |
| 18–29 | 1805 (46.7) | 994 (43.9) | 811 (50.7) |  |
| 30–39 | 1252 (32.4) | 715 (31.6) | 537 (33.6) |  |
| 40–49 | 531 (13.7) | 398 (17.6) | 133 (8.3) |  |
| >50 | 277 (7.2) | 159 (7.0) | 118 (7.4) |  |
| **HBV diagnosis date, years** | | | | <0.001 |
| 1990–1999 | 861 (22.3) | 469 (20.7) | 392 (24.5) |  |
| 2000–2009 | 1554 (40.2) | 895 (39.5) | 659 (41.2) |  |
| 2010–2015 | 1450 (37.5) | 902 (39.8) | 548 (34.3) |  |
| **Education level (years in school), years** | | | | <0.001 |
| ≤9 | 1395 (36.1) | 778 (34.3) | 617 (38.6) |  |
| 10–12 | 1246 (32.2) | 756 (33.4) | 490 (30.6) |  |
| >13 | 858 (22.2) | 567 (25.0) | 291 (18.2) |  |
| Missing | 366 (9.5) | 165 (7.3) | 201 (12.6) |  |
| **African region of birth** | | | | 0.90 |
| Northern | 261 (6.8) | 152 (6.7) | 109 (6.8) |  |
| Eastern | 2478 (64.1) | 1455 (64.2) | 1023 (64.0) |  |
| Middle | 240 (6.2) | 152 (6.7) | 94 (5.9) |  |
| Western | 874 (22.6) | 505 (22.3) | 369 (23.1) |  |
| Southern | 12 (0.3) | 8 (0.4) | 4 (0.3) |  |
| **Co-morbidities** | | | |  |
| HCV co-infection | 194 (5.0) | 130 (5.7) | 64 (4.0) | 0.02 |
| HDV co-infection | 105 (2.7) | 63 (2.8) | 42 (2.6) | 0.77 |
| HIV co-infection | 126 (3.3) | 75 (3.3) | 51 (3.2) | 0.84 |
| Alcohol overconsumption | 93 (2.4) | 75 (3.3) | 18 (1.1) | <0.001 |
| Diabetes mellitus | 326 (8.4) | 221 (9.8) | 105 (6.6) | <0.001 |
| Obesity | 28 (0.7) | 3 (0.1) | 25 (1.6) | <0.001 |
| Drug misuse | 60 (1.6) | 47 (2.1) | 13 (0.8) | 0.002 |
| **Co-medications** | | | |  |
| Interferon therapy | 39 (1.0) | 32 (1.4) | 7 (0.4) | 0.003 |
| Nucleos(t)ide analogues | 260 (6.7) | 178 (7.9) | 82 (5.1) | <0.001 |
| Statin | 224 (5.8) | 157 (6.9) | 67 (4.2) | <0.001 |
| Aspirin | 135 (3.5) | 81 (3.6) | 54 (3.4) | 0.74 |
| **Follow-up time, years** | | | |  |
| Mean (SD) | 12.4 (6.7) | 12.0 (6.6) | 13.1 (6.8) | <.0001 |
| *Abbreviations: CHB=chronic HBV; SD= standard deviation; HCV=hepatitis C virus; HDV=hepatitis Delta virus;* **^¶^***=The age or year at first immigration event, if >one event of immigration; *=HIV and or HCV and/or HDV and/or DM.* | | | | |

| **Table 2: Incidence rate ratios (IRR) for HCC derived from Poisson regression models presenting the association of variables in a chronic hepatitis B cohort of persons with African origin.** | | | | | | | | | |
| --- | --- | --- | --- | --- | --- | --- | --- | --- | --- |
|  | **Univariable** | | | **Base model (multivariable)** | | | **Adjusted estimates^#^** | | |
| **Predictors** | **IRR** | **95% CI** | **p** | **IRR** | **95% CI** | **p** | **IRR** | **95% CI** | **p** |
| **Base Model** | | | | | | | | | |
| Age (centered) | 1.07 | 1.04-1.09 | 0* | 1.12 | 1.07-1.18 | 0* | - | - | - |
| Men | 2.66 | 1.21-6.68 | 0.02* | 7.13 | 2.08-42.31 | 0.01* | - | - | - |
| Age * men | - | - | - | 0.94 | 0.88-0.99 | 0.03* | - | - | - |
| **Co-morbidities** | | | | | | | | | |
| HCV co-infection | 4 | 1.49-9.14 | 0.002* | - | - | - | **2.75** | **1.01-6.37** | **0.03*** |
| HDV co-infection | 4.27 | 1.02-12 | 0.02* | - | - | - | **4.47** | **1.06-12.84** | **0.02*** |
| HIV co-infection | 1.99 | 0.32-6.6 | 0.3 | - | - | - | 2.01 | 0.32-6.73 | 0.3 |
| Diabetes mellitus | 1.81 | 0.61-4.33 | 0.2 | - | - | - | 0.97 | 0.32-2.43 | >0.9 |
| **African region of origin (vs Eastern)** | | | | | | | | | |
| Middle | 2.73 | 0.79-7.39 | 0.07* | - | - | - | **3.82** | **1.08-10.65** | **0.02*** |
| Northern | 1.6 | 0.37-4.76 | 0.5 | - | - | - | 1.73 | 0.4-5.3 | 0.4 |
| Western | 1.15 | 0.44-2.66 | 0.8 | - | - | - | 1.77 | 0.66-4.32 | 0.2 |
| *CI= Confidence Intervals; p=p-value.* **^#^***Adjusted estimates are univariable estimates adjusted for the Base Model.* **P-values < 0.05.* | | | | | | | | | |

| ***Table S1: Baseline characteristics of African-born individuals with chronic hepatitis B without cirrhosis per African region of birth*** | | | | | | |
| --- | --- | --- | --- | --- | --- | --- |
|  | **Middle** | **Eastern** | **Northern** | **Southern** | **Western** | **P-value** |
| **Total, n (%)** | 240 | 2478 | 261 | 12 | 874 | <0.001 |
| Men, n (%) | 146 (60.8) | 1455 (58.7) | 152 (58.2) | 8 (66.7) | 505 (57.8) | 0.15 |
| **Age at start of follow-up** | | | | | |  |
| All, mean (SD) | 29.8 (9.1) | 32.7 (12.0) | 35.3 (9.5) | 32.0 (11.1) | 29.9 (9.6) | <0.001 |
| Men, mean (SD), years | 30.2 (8.8) | 32.8 (11.7) | 36.9 (9.7) | 34.9 (12.1) | 31.0 (10.5) | <0.001 |
| Women, mean (SD), years | 29.4 (9.4) | 32.6 (12.3) | 33.0 (8.8) | 26.1 (6.1) | 28.3 (8.0) |  |
| **Age groups (years), n (%)** | | | | | | <0.001 |
| 20–29 | 125 (52.1) | 1139 (46.0) | 78 (29.9) | 6 (50.0) | 457 (52.3) |  |
| 30–39 | 90 (37.5) | 759 (30.6) | 113 (43.3) | 3 (25.0) | 287 (32.8) |  |
| 40–49 | 16 (6.7) | 359 (14.5) | 46 (17.6) | 3 (25.0) | 107 (12.2) |  |
| ≥50 | 9 (3.8) | 221 (8.9) | 24 (9.2) | 0 | 23 (2.6) |  |
| **HBV diagnosis date/date of matching, n (%)** | | | | | | <0.001 |
| 1990–1999 | 24 (10.0) | 594 (24.0) | 65 (24.9) | 1 (8.3) | 177 (20.3 |  |
| 2000–2009 | 131 (54.6) | 907 (36.46) | 92 (35.2) | 7 (58.3) | 417 (47.7) |  |
| 2010–2015 | 85 (35.4) | 977 (39.4) | 104 (39.8) | 4 (33.3) | 280 (32.0) |  |
| **Education level (years in school), n (%)** | | | | | | <0.001 |
| = <9 | 41 (17.1) | 1056 (42.6) | 68 (26.1) | 1 (8.3) | 229 (26.2) |  |
| 10–12 | 71 (29.6) | 718 (29.0) | 101 (38.7) | 5 (41.7) | 351 (40.2) |  |
| >=13 | 114 (47.5) | 410 (16.5) | 79 (30.3) | 5 (41.7) | 250 (28.6) |  |
| Unknown | 14 (5.8) | 294 (11.9) | 13 (5.0) | 1 (8.3) | 44 (5.0) |  |
| **Co-morbidities, n (%)** | | | | | |  |
| HCV | 19 (7.9) | 113 (4.6) | 19 (7.3) | 1 (8.3) | 42 (4.8) | 0.08 |
| HDV | 6 (2.5) | 61 (2.5) | 10 (3.8) | 0 | 28 (3.2) | 0.53 |
| HIV | 10 (4.2) | 71 (2.9) | 3 (1.1) | 5 (41.7) | 37 (4.2) | <0.001 |
| Alcohol overconsumption | 2 (0.8) | 54 (2.2) | 11 (4.2) | 1 (8.3) | 25 (2.9) | 0.06 |
| DM | 15 (6.3) | 225 (9.1) | 36 (13.8) | 0 | 50 (5.7) | <0.001 |
| Drug misuse | 0 | 29 (1.2) | 11 (4.2) | 0 | 20 (2.3) | <0.001 |
| **Therapies, n (%)** | | | | | |  |
| Interferon | 0 | 20 (0.8) | 4 (1.5) | 0 | 15 (1.7) | 0.07 |
| Nucleos(t)ide analogues | 25 (10.4) | 130 (5.2) | 29 (11.1) | 1 (8.3) | 75 (8.6) | <0.001 |
| Aspirin | 8 (3.3) | 70 (2.8) | 17 (6.5) | 1 (8.3) | 39 (4.5) | 0.02 |
| Statin | 10 (4.2) | 146 (5.9) | 29 (11.1) | 0 | 39 (4.5) | 0.001 |
| **Follow-up time in years** | | | | | |  |
| Mean (SD) | 11.1 (5.5) | 12.4 (6.9) | 13.1 (5.3) | 10.4 (5.3) | 12.7 (6.2) | 0.02 |
| *Abbreviations: CHB=chronic hepatitis B; SD= standard deviation; IQR=interquartile range; HCV=hepatitis C virus; HDV=hepatitis Delta virus; HIV=human immunodeficiency virus;DM=diabetes mellitus. Numbers are presented as frequencies and % percentages within column)* | | | | | | |

| ***Table S2.* *Baseline characteristics of African-born men with CHB, and in comparator cohorts from the same area of origin and the general population without HBV infection.*** | | | | |
| --- | --- | --- | --- | --- |
| **Characteristics** | **Men with CHB (n, %)** | **Cohort from the same area of origin (n, %)** | **Cohort from the general population (n, %)** | **P-value** |
| **Total*** | 2266 (58.6) | 5048 (59.5) | 23,188 (59.0) |  |
| **Age at start of follow-up (years)** | | | |  |
| All, mean (SD) | 32.5 (11.2) | 32.9 (10.9) | 32.9 (11.4) | 0.16 |
| **Age groups, n (%)** |  |  |  | 0.20 |
| 18–29 | 994 (43.9) | 2116 (41.9) | 9877 (42.6) |  |
| 30–39 | 715 (31.6) | 1652 (32.7) | 7306 (31.5) |  |
| 40–49 | 398 (17.6) | 914 (18.1) | 4149 (17.9) |  |
| >50 | 159 (7.0) | 366 (7.3) | 1856 (8.0) |  |
| **HBV diagnosis date (years), n (%)/matching date for comparators** | | | | <0.001 |
| 1990–1999 | 469 (20.7) | 1034 (20.5) | 4762 (20.5) |  |
| 2000–2009 | 895 (39.5) | 1789 (35.4) | 9176 (39.6) |  |
| 2010–2015 | 902 (39.8) | 2225 (44.1) | 9250 (39.9) |  |
| **Education level (years in school), n (%)** | | | | <0.001 |
| ≤9 | 778 (34.3) | 1613 (32.0) | 4095 (17.7) |  |
| 10–12 | 756 (33.4) | 1882 (37.3) | 10,109 (43.6) |  |
| >13 | 567 (25.0) | 1186 (23.5) | 7699 (33.2) |  |
| Missing | 165 (7.3) | 367 (7.3) | 1285 (5.5) |  |
| **African region of birth, n (%)** | | | | 0.01 |
| Northern | 152 (6.7) | 346 (6.9) | 185 (0.8) |  |
| Eastern | 1455 (64.2) | 3643 (72.2) | 862 (3.7) |  |
| Middle | 146 (6.4) | 202 (4.0) | 42 (0.2) |  |
| Western | 505 (22.3) | 847 (16.8) | 93 (0.4) |  |
| Southern | 8 (0.4) | 8 (0.2) | 6 (0.03) |  |
| Other | - | 2 (0.0) | 22,000 (94.9) |  |
| **Co-morbidities, n (%)** | | | |  |
| HCV co-infection | 130 (5.7) | 43 (0.9) | 11 (0.05) | <0.001 |
| HDV co-infection | 63 (2.8) | - | - |  |
| HIV co-infection | 75 (3.3) | 45 (0.9) | 31 (0.1) | <0.001 |
| Alcohol overconsumption | 75 (3.3) | 130 (2.6) | 746 (3.2) | 0.05 |
| DM | 221 (9.8) | 477 (9.4) | 1143 (4.9) | <0.001 |
| Drug misuse | 47 (2.1) | 95 (1.9) | 226 (1.0) | <0.001 |
| **Co-medications, n (%)** | | | |  |
| Interferon therapy | 32 (1.4) | 8 (0.2) | 5 (0.02) | <0.001 |
| Nucleos(t)ide analogues | 178 (7.9) | 8 (0.2) | 5 (0.02) | <0.001 |
| Statin | 157 (6.9) | 417 (8.3) | 2025 (8.7) | 0.01 |
| Aspirin | 81 (3.6) | 185 (3.7) | 1075 (4.6) | 0.001 |
| **Follow-up time (years)** | | | |  |
| Mean (SD) | 11.9 (6.6) | 11.8 (6.7) | 12.0 (6.6) | 0.04 |
| *Abbreviations: *Compared to the total respective cohort; CHB=chronic HBV; SD= standard deviation; HCV=hepatitis C virus; HDV=hepatitis Delta; HIV=human immunodeficiency virus; Diabetes mellitus. ^=HIV and or HCV and/or HDV and/or DM. Numbers are rounded to the 2^nd^ decimal. Frequencies are presented as numbers and percentages within column.* | | | | |
|  | | | | |

| ***Table S3: Characteristics and survival of African-born persons with chronic hepatitis B with hepatocellular carcinoma, by sex.*** | | | | |
| --- | --- | --- | --- | --- |
|  | **All** | **Men** | **Women** | **P-value** |
| Total, n (%) by row | 31 (100) | 24 (77.4) | 7 (22.6) | <0.001 |
| **Age at immigration, mean (SD), years** | 35.5 (16.4) | 30.4 (12.1) | 53.2 (18.0) | 0.22 |
| **Age at HCC diagnosis, mean (SD), years** | 51.4 (16.6) | 46.8 (14.7) | 67.0 (13.7) | 0.003 |
| **Date of HBV diagnosis, n (%)** | | | | 0.37 |
| 1990–1999 | 10 (32.3) | 8 (33.3) | 2 (28.6) |  |
| 2000–2009 | 17 (54.8) | 14 (58.3) | 3 (42.9) |  |
| 2010–2015 | 4 (12.9) | 2 (8.3) | 2 (28.6) |  |
| **Level of education, n (%), years in school** | | | | 0.02 |
| ≤9 | 4 (12.9) | 2 (8.3) | 2 (28.6) |  |
| 10–12 | 12 (38.7) | 11 (45.8) | 1 (14.3) |  |
| >13 | 8 (26.8) | 8 (33.3) | 0 |  |
| Missing | 7 (22.6) | 3 (12.5) | 4 (57.1) |  |
| **African region of birth, n (%)** | | | | 0.21 |
| Northern | 3 (12.9) | 3 (12.5) | 0 |  |
| Eastern | 17 (54.8) | 11 (45.8) | 6 (85.7) |  |
| Middle | 4 (12.9) | 3 (11.1) | 1 (14.3) |  |
| Western | 7 (22.6) | 7 (29.2) | 0 |  |
| **Comorbidities, n (%)** | | | |  |
| HCV | 6 (19.4) | 4 (16.7) | 2 (28.6) | 0.49 |
| HDV | 3 (9.7) | 3 (12.5) | 0 | 0.20 |
| HIV | 2 (6.5) | 2 (8.3) | 0 | 0.30 |
| DM | 5 (16.1) | 5 (20.8) | 0 | 0.56 |
| Cirrhosis diagnosis during follow-up | 15 (48.4) | 11 (45.8) | 4 (57.1) | 0.59 |
| Received IFN | 2 (6.5) | 2 (8.3) | 0 | 0.43 |
| Received nucleos(t)ide analogues | 15 (48.4) | 11 (45.8) | 4 (57.1) | 0.59 |
| Received liver transplant after HCC diagnosis | 2 (6.5) | 1 (4.2) | 1 (14.3) | 0.33 |
| **Survival excluding those diagnosed from Death Register (n=13)** | | | | |
| Survival at 1 year, n (%) | 8 (25.8) | 5 (20.8) | 3 (42.9) | 0.01 |
| Survival time, median (IQR), months | 8.2 (2.9-89.2) | 6.7 (2.0-82.3) | 64.4 (17.1-104.1) | 0.29 |
| **Follow-up time in years** | | | | |
| Median (IQR) | 9.2 (3.9–14.3) | 8.5 (3.5–13.7) | 9.2 (6.0–15.8) | 0.97 |
| *Abbreviations: n= number; HCC= hepatocellular carcinoma; SD= standard deviation; IQR=interquartile range; HCV=hepatitis C virus; HDV=hepatitis Delta virus; HIV=human immunodeficiency virus; DM=diabetes mellitus;IFN=interferon; na=not applicable. Numbers are rounded to the 2nd decimal. Proportions are presented by column. NB: none of persons with CHB who developed HCC had a record of alcohol overconsumption or obesity.* | | | | |

| ***Table S4: Characteristics and survival of persons with chronic hepatitis B with HCC per African region of birth*** | | | | | |
| --- | --- | --- | --- | --- | --- |
|  | **Middle** | **Eastern** | **Western** | **Northern** | **P-value** |
| **Total, n (%)** | 240 (6.2) | 2478 (64.1) | 874 (22.6) | 261 (6.8) | <0.001 |
| Number of persons with HCC, n (%) | 4 (1.7) | 17 (0.7) | 7 (0.8) | 3 (1.2) | 0.03 |
| Men, n (%) | 3 (75) | 11 (64.7) | 7 (100) | 3 (100) |  |
| Women, n (%) | 1 (25) | 6 (35.3) | 0 | 0 |  |
| **Age at HCC diagnosis (years)** |  | | | |  |
| All, median (IQR) | 36.9 (28.7–69.3) | 49.5 (38.0–67.6) | 54.9 (35.0–62.9) | 59.7 (36.8–59.7) | 0.64 |
| Men, mean (SD) | 34.1 (9.2) | 43.9 (12.8) | 51.9 (13.3) | 58.5 (21.2) | (0.05) ** |
| Women, mean (SD) | 77.5 | 65.2 (14.1) | 0 | 0 | 0.57 |
| **Date of HBV diagnosis, n (%)** | | |  |  | 0.51 |
| 1990–1999 | 0 | 6 (35.3) | 2 (28.6) | 2 (66.7) |  |
| 2000–2009 | 3 (75.0) | 9 (52.9) | 4 (57.1) | 1 (33.3) |  |
| 2010–2015 | 1 (25.0) | 2 (11.8) | 1 (14.3) | 0 |  |
| **Level of education (years in school), n (%)** | | | | | 0.70 |
| ≤ 9 | 1 (25) | 3 (17.6) | 0 | 0 |  |
| 10–12 | 1 (25) | 5 (29.4) | 5 (71.4) | 1 (33.3) |  |
| > 13 | 1 (25) | 5 (29.4) | 1 (14.3) | 1 (33.3) |  |
| Missing | 1 (25) | 4 (23.5) | 1 (14.3) | 1 (33.3) |  |
| **Co-morbidities, n (%)** |  | | | |  |
| HCV | 0 | 5 (29.4) | 0 | 1 (33.3) | 0.11 |
| HDV | 1 (25.0) | 1 (5.9) | 0 | 1 (33.3) | 0.28 |
| HIV | 0 | 1 (5.9) | 1 (14.3) | 0 | 0.68 |
| DM | 1 (25.0) | 1 (5.9) | 1 (14.3) | 2 (66.7) | 0.13 |
| Cirrhosis diagnosis during follow-up | 3 (75.0) | 8 (47.1) | 1 (14.3) | 3 (100) | 0.03 |
| Received IFN therapy | 0 | 1 (5.9) | 1 (14.3) | 0 | 0.75 |
| Received nucleos(t)ide analogues | 3 (75.0) | 7 (41.2) | 3 (42.9) | 2 (66.7) | 0.57 |
| Received liver transplant after HCC diagnosis | 0 | 2 (11.7) | 0 | 0 |  |
| **Survival excluding those diagnosed from Death Register (n=13), n (%)** | | | | | |
| Survival at 1 year | 0 | 7 (41.2) | 1 (14.3) | 0 | 0.42 |
| Survival time^&^, median (IQR), months | 0.0 | 7.7 (0.0-81.6) | 0.5 (0.0-6.5) | 0.5 (0.0-0.5) | 0.01 |
| **Follow-up time in years** |  | | | |  |
| Median (IQ) | 3.01 (1.2–4.4) | 9.4 (5.6–11.7) | 10.0 (4.8–18.7) | 14.3 (3.9–14.3) | 0.002 |
| *Abbreviations: n=number; HCC= hepatocellular carcinoma; SD= standard deviation; IQR=interquartile range; HCV=hepatitis C virus; HDV=hepatitis Delta virus; HIV=human immunodeficiency virus; DM=diabetes mellitus;na=not applicable. * Including patients who underwent liver transplantation. NB: none of the persons with CHB who developed HCC had a record of alcohol overconsumption or obesity. **Middle vs Northern Africa was significant younger (p=0.04), Middle vs Western Africa was marginally significant at (p=0.05)* | | | | | |
