# Supplementary material for: Age-specific and sex-specific risks for HCC in African-born persons with chronic hepatitis B without cirrhosis
Source: Hepatol Commun. 2023 Dec 1;7(12):e0334. doi: 10.1097/HC9.0000000000000334 (PMC10697596; doi:10.1097/HC9.0000000000000334)
Supplement: SUPPLEMENTARY MATERIAL [file hc9-7-e0334-s002.docx]

**HEP4-23-0604**

**Age and sex-specific risks for hepatocellular carcinoma in African-born persons with chronic hepatitis B without cirrhosis**

Authors:

Habiba Kamal^1,2^, Michael Ingre^2,3^, Per Stål^2,4^, Gabriel Westman^5^, Daniel Bruce^6^, Heiner Wedemeyer^7^, Ann-Sofi Duberg^8^* and Soo Aleman^1,2^*

*= equal contribution

**Affiliations:**

1. Department of Infectious Diseases, Karolinska University Hospital, Stockholm, Sweden
2. Department of Medicine Huddinge, Karolinska Institute, Stockholm, Sweden
3. Centre for Bioinformatics and Biostatistics, Karolinska Institute, Stockholm, Sweden
4. Department of Upper GI Diseases, Karolinska University Hospital, Stockholm, Sweden
5. Department of Medical Sciences, Section of Infectious diseases, Uppsala University, Uppsala Sweden
6. SDS Life Science, Stockholm, Sweden
7. Department of Gastroenterology and Hepatology, University of Hannover, Germany
8. Department of Infectious Diseases, Örebro University Hospital, Örebro, Sweden

**Short Title:** Risk of HCC in African-born persons with CHB.

**Corresponding author:**

Habiba Kamal, M.D., M.Sc.

Department of Infectious Diseases, I53

Karolinska University Hospital, Huddinge

Stockholm, Sweden

Tel.: +46-769476182

Email : Habiba.khodir.kamal@ki.se

Abstract : 272

Abstract -and body text:4216

References: 40 references.

Tables: 2 main, 4 supplementary.

Figures: 3 main, 2 supplementary

Keywords: HCC, HBV, surveillance, cirrhosis, risk

Conflict of Interest: Habiba Kamal received grants from Gilead. Per Stal is on the speakers’ bureau for Roche, Albireo and Eisai. Heiner Wedemeyer consults for AstraZenece, Eisai and MSD. Ann-Sofi Duberg advises and is on the speakers’ bureau for Gilead. Soo Aleman is on the speakers’ bureau for Gilead.

**Abstract**

**Introduction:** The international recommendations of HCC surveillance for African-born persons with chronic hepatitis B (CHB) without cirrhosis are divergent, probably due to scarce data on incidence rate (IR) for HCC.

**Methods:** We assembled a cohort with prospectively collected data of Swedish residents of African origin with diagnosed CHB without cirrhosis at baseline from 1990- 2015. Data from nationwide registers were used to calculate the sex-specific IR and IR ratio (IRR) in relation to age, comorbidities and birth region, using a generalized linear model with a log-link function and Poisson distribution.

**Results:** Among 3,865 African-born CHB persons without cirrhosis at baseline, 31 (0.8%; 77.4% men) developed HCC during a median of 11.1 years of follow-up, with poor survival after HCC diagnosis. The mean age at HCC diagnosis was 46.8 (SD±14.7; range 23-79) in men. HCC IR exceeded the recommended surveillance threshold 0.2%/year at age 54 and 59 years in men and women, respectively, and at age 20-40 years if present HCV or HDV co-infection. African-born men with CHB had IRR of 10.6 (95% CI 4.4-31.5) for HCC compared to matched African-born peers without CHB, and an IRR of 35.3 (95% CI 16.0-88.7) compared to a matched general population.

**Conclusions:** African-born men with CHB without cirrhosis reached an IR of 0.2%/year between 50-60 years, while younger ages if present HCV or HDV co-infection. Our findings need further confirmation, and new cost-effectiveness analyses specific for young populations are needed, to provide personalized and cost-effective HCC surveillance.

**Introduction**

Hepatocellular carcinoma (HCC) is the second leading cause of cancer-related mortality among men and the 6^th^ among women in Africa, with overall 80% of HCC being attributed to hepatitis B virus (HBV) infection [1]. It is estimated that 11 million African-born migrants reside in Europe, five million in Asia, and three million in Northern America [2]. This has changed the epidemiology of chronic hepatitis B (CHB) in several low-endemic countries hosting African migrants [3]. Diagnosis of HCC at an advanced stage has been previously reported in young men of African origin with CHB [4,5].

Surveillance for HCC with biannual ultrasound +/- alfa-fetoprotein is currently recommended for persons at high risk for HCC, to detect tumors at early and curable stages [6]. Current guidelines have used a cost-effectiveness threshold incidence for surveillance of 0.2% per year for CHB patients without liver cirrhosis and 1.5% for those with cirrhosis, for recommendations on when to start surveillance for HCC [7,8]. The knowledge about the incidence rates (IR) of HCC in African-born immigrants with CHB and without cirrhosis per age and sex is though scarce. Our research group in a previous study showed that the annual risk for HCC in men with CHB from Sub-Saharan Africa, including both those with cirrhosis and no cirrhosis, exceeded 0.2%/year from age group 50-59 years [9]. The paucity of incidence rate data for HCC is a probable reason for discordant international recommendations regarding from which age to start HCC surveillance in non-cirrhotic persons with CHB of African origin, especially for men [5].

The American Association for the Study of Liver Disease (AASLD) recommends HCC surveillance for African or African-American men of age ≥40 years [10]. In contrast, the Asian Pacific Association for the Study of the Liver (APASL) and the Canadian Association for the Study of the Liver (CASL) recommend surveillance of persons with CHB and African origin from 20 years of age, regardless of sex [11,12]. The Chinese guideline recommends surveillance of all persons with CHB, including non-cirrhotic persons regardless of age [13]. The European Association for the Study of the Liver (EASL) does not take into account any ethnicity but recommends HCC surveillance based on the risk score of Platelet-Age-Gender–HBV (PAGE-B) [14]. This risk score has been validated in Caucasian and Asian populations, but not in an African population [15,16]. The Swedish national guideline recommends HCC surveillance for CHB in men >40 years and women >50 years with African origin, especially if other risk factors are present [17].

In this study, we included a nationwide cohort of 3,865 African-born individuals with CHB without cirrhosis and investigated the IR of HCC in relation to age, sex, co-morbidities, and region of birth. The risk in the study population was compared with the risk in individuals without CHB from the same country of origin, and from the general population, matched for age, sex, and county of residence.

**Materials and Methods**

*Study population*

A nationwide dataset including all persons who had been diagnosed with HBV infection through HBsAg positivity (n=41,796) from 1^st^ of January 1990 to 31^st^ December 2015 was constructed, using data from the National Surveillance Register SmiNet at the Public Health Agency (Flow chart in Figure 1). Only African-born persons with CHB and residency in Sweden at date of diagnosis were included, then categorized by country of birth into Northern, Eastern, Middle, Western and Southern Africa, according to United Nations regional geographical definition [18]. We included persons from the whole African continent, extended the follow-up time until the end of 2019 compared to our previous study [9], and excluded those with cirrhosis prior to or within 6 months after CHB diagnosis. Cirrhosis diagnosis was ascertained from the Patient Register using International Classification of Diseases (ICD) codes (ICD-9 and -10) for cirrhosis at hospital discharge or outpatient specialty care, as previously described [19].

*Linkage to national registers for outcomes*

We linked the dataset of persons with CHB to validated national registries, the Patient Register (PR), Cause of Death Register (DR), Cancer Register (CR), and Prescribed Drug Register (PDR) at the National Board of Health and Welfare, described in our previous study [9]. The PR contains prospectively updated national data of all hospitalizations including liver transplantations, discharge diagnoses (since 1964), and diagnoses from outpatient specialist care (since 2001). The diagnoses are recorded with ICD-codes in well-validated registries. The PDR has prospectively recorded all dispensed prescriptions, including antivirals against HBV infection, i.e., nucleos(t)ide analogs (NA) or pegylated interferon (IFN), from Swedish pharmacies since 2005.

Persons with ≤6 months of follow-up, and persons who had at least two episodes (to rule out misclassification) with diagnoses of other liver diseases as hereditary hemochromatosis, autoimmune and parasitic causes, liver cancer, or any diagnoses of cancer except non-melanoma skin cancer, and liver transplantation, prior to or within 6 months after CHB diagnosis were excluded (n=219). The index date, i.e., the start of follow-up at baseline, was set to the date of HBV notification to the Public Health Agency, plus a time window of six additional months, to avoid surveillance bias. The final cohort for the analysis constituted of 3,865 persons with CHB.

Demographics and other parameters, including sex, age at HBV diagnosis, alcohol overconsumption, co-morbidities such as diabetes mellitus (DM), obesity, hepatitis D virus (HDV), hepatitis C virus (HCV), and human immunodeficiency virus (HIV) co-infections, as well as HBV treatment were collected from the mentioned registers through ICD and ATC (Anatomical Therapeutic Chemical Classification system) codes.

*Outcomes*

The primary outcome was liver cancer (ICD codes as published in a prior work [19]) primarily retrieved from the CR, and the DR for combining data increases the coverage [20]. Liver cancer is referred to as HCC in this study, as most liver cancers are HCC in a cohort with CHB [20]. The study participants were followed until the first recorded date of HCC, death, liver transplantation, or 31^st^ December 2019, whichever occurred first.

*Comparison cohorts*

Two matched comparison cohorts without CHB diagnosis were obtained from Statistics Sweden. The matching date was the date of HBV notification and the comparators had to be alive and reside in Sweden at that date. First cohort: each subject with CHB was matched with up to three individuals (when possible) on birth year, sex, county of residence at notification, and country of origin (n=8,488). Second cohort: each subject with CHB was matched on birth year, sex, and county of residence in Sweden with up to 10 individuals from the Swedish general population (n=39,267).

*Ethical permit*

The Regional Ethical Review Board in Stockholm, Sweden approved the study (Dnr 2015/1282-31). As this study is register-based using pseudonymized data, informed consent from the participants was waived.

**Statistical analysis**

Continuous variables were presented as mean (standard deviation [SD]) when normally distributed, and as median (interquartile range [IQR]) when skewed distribution. Student-t and Mann-Whitney tests were used for the comparison of normal and skewed continuous variables, respectively. Categorical variables were presented as frequencies and proportions and were compared using Chi-Square test or Fisher’s exact test, whenever appropriate.

Incidence rates (IR) of HCC during follow-up were calculated as the number of new HCC diagnoses divided by the sum of person-years at risk and were reported as events per 100 person-years (e.g., [number of new-onset HCC/person-years] *100) with 95% confidence intervals (95% CI). To calculate IR and incidence rate ratios (IRR) a generalized linear model was applied to data, using a log-link function together with a Poisson error distribution and an offset with the logarithm of follow-up time constrained to a coefficient of 1. The model aimed to calculate the sex-specific age when IR would cross the currently recommended surveillance threshold of 0.2%/year. To that end, a base model was fitted with HCC as the dependent variable and independent variables indicating sex and age on a continuous scale (centered around the mean) together with the interaction age*sex. All terms in the model were significant with CIs that did not include unity (see results below). Sensitivity analyses indicated that the base model (Akaike Information Criterion; AIC=371) provided superior fit over a model with four discrete age groups (AIC=384) and that adding polynomials (AIC=373) or natural splines (AIC=373) to the model to account for potential nonlinearity of age did not improve fit over the base model. The base model was used to calculate the sex-specific age when IR crossed above the surveillance threshold and was also used to test if adding co-morbidities or regions of origin could add to the prediction.

All P-values were two-tailed and statistical significance was set to P value <0.05. Analyses were performed using SAS® (version 9.4, SAS Institute Inc., Cary, NC, USA), SPSS IBM Statistics (version 28.0), and R version 4.2.2.

**Results**

*Baseline characteristics*

The baseline characteristics of the 3,865 African-born persons with CHB and without liver cirrhosis at baseline are presented in Table 1. The mean (±SD) age at HBV diagnosis was 32.1 (±11.2) years. Men constituted 58.6% of the cohort. The predominant area of origin was Eastern Africa (64.1%), followed by Western Africa (22.6%). Men had significantly more frequent HCV co-infection, alcohol overconsumption, DM, and drug misuse at baseline, and were more frequently prescribed anti-HBV therapies than women (all P=<0.05).

The characteristics of persons with CHB by African region of birth are presented in Table S1. Middle, Northern- and Southern African persons had significantly more frequent HCV co-infections, compared to peers from Eastern and Western Africa (P=<0.001). Characteristics of the two comparator groups (men) are presented in Supplementary Table S2.

*Characteristics of persons who developed HCC.*

Thirty-one (0.8%) persons developed HCC during a median follow-up (IQR) of 11.1 (6.6-17.6) years, corresponding to 48,066 person-years. The majority were men (n=24, 77.4%) (Table S3). Seventy-four persons (1.9%) were diagnosed with cirrhosis during follow-up. The mean±SD age at HCC diagnosis was 51.4±16.6 years, with a significantly lower age in men (mean 46.8±14.7; range 23-79 years) than in women (mean 67±13.7; range 48-82 years) (P=0.03) (Figure 2). The mean (±SD) age at immigration for persons who developed HCC was significantly higher at 35.5 (±16.4) years, compared to 27.5 (±11.6) years for those who did not develop HCC (p=0.01). For men, the difference was though not statistically significant when comparing the age of immigration in those with and without HCC (30.4 vs 27.5 years of age, p=0.22).

Fifteen of persons with HCC (48%) had received cirrhosis diagnosis prior to HCC diagnosis. However, nine (60%) of them had received cirrhosis diagnosis ≤6 months prior to HCC diagnosis, including eight (8/15, 53%) with ≤3 months. Fifteen persons (48%) received NA prior to HCC diagnosis for a median duration of 11.0 months (IQR, 6.7-15.3).

Development of HCC <40 years of age was seen in 10 men (10/24, 42%). Among them, all five (50%) with liver cirrhosis at the time of HCC, had received the cirrhosis diagnosis ≤3 months prior to HCC diagnosis. The characteristics of persons who developed HCC, and sub-grouped by area of origin are shown in Table S4.

*Risk for development of HCC*

To estimate the age and sex-specific risk of HCC a base model was fitted, showing a significant IRR for men (IRR=7.13; 95% CI 2.08-42.31), age in years (IRR=1.12, 95% CI 1.07-1.18) and the interaction age* sex (IRR=0.94; 95% CI 0.88-0.99). This indicates that men were at higher risk for HCC than women, and that the risk increases with age but that this increase was attenuated with older age in men (Table 2). Figure 3 shows the predicted IR, illustrating also that the increase of risk in men was most pronounced at younger ages.

Overall, men had an IR of 0.09/100PYs (95% CI 0.06-0.13), while women had IR of 0.03/100PYs (95% CI 0.01-0.07). Men exceeded the currently recommended threshold for HCC surveillance (0.2%/year) at 54 years of age (IR=0.20/100PYs, 95% CI 0.10-0.40), while women did so at 59 years of age (IR=0.21/100PYs, 0.10-0.45) (Figure 3).

Table 2 shows the HCC risk estimates of co-morbidities with HCV-, HDV- or HIV co-infection and diabetes, adjusted for the predictors of the base model (age, sex, and age*sex) indicating increased risk for co-infection with HCV (IRR=2.75, 95% CI 1.01-6.37) and HDV (IRR=4.47; 95% CI 1.06-12.84) but not for HIV (IRR=2.0, 95% CI 0.32-6.73) or diabetes (IRR=0.97, 95% CI 0.32-2.43). Figure S1 indicates that the increased risk with HCV or HDV co-infection placed men with these co-infections above the recommended surveillance threshold at the age of 33 years (IR=0.20/100PYs, 95% CI 0.08-0.48) and 20 years (IR=0.21/100PYs, 95% CI 0.05-0.75), respectively. Women reached the threshold at 51 and 46 years of age, respectively.

A similar analysis on the region of origin (Table 2) showed that persons from Middle Africa have an increased risk of HCC (IRR=3.82;95% CI 1.08-10.65) compared to the Eastern region. The risk was similar when comparing those from Northern (IRR)=1.73; 95% CI 0.40-5.30) or Western regions (IRR=1.77; 95% CI 0.66-4.32) to Eastern Africa. Predictions of HCC per region can be found in Figure S1b.

The incidence rate of HCC per sex for persons with CHB and comparators are shown in Figure S2.

*Survival of persons with CHB who developed HCC.*

Twenty-three (74%) African-born persons with CHB and HCC died during follow-up. Diagnosis of HCC was recorded at the same date as for death ~~as~~ in 42 % (13/31, 10 men and 3 women), with the retrieval of data from the DR. For the other, the median (IQR) survival time after HCC diagnosis was 8.2 (2.9-89.2) months, with similar survival time in men compared to women (Table S4). The median (IQR) survival time for men <40 years at HCC diagnosis was 4.4 (1.6-39.9) months, which was numerically lower than for men >40 years with 25.6 months (P> 0-05). One man (4.2%) and one woman (14.3%) with an HCC diagnosis received a liver transplant, respectively.

*Risks of HCC compared to comparators.*

African-born men with CHB had 10.6-times the risk to develop HCC compared to matched African-born peers without CHB (IRR=10.6, 95% CI 4.4-31.5) and 35.3-times the risk to develop HCC compared to the matched general population (IRR=35.3, 95% CI 16.0-88.7). For African-born men with age <40 years, the corresponding figures were 30.3-times the risk compared to African-born peers without CHB (IRR=30.3; 95% CI 4.0-230.5) and 141-times the risk to develop HCC compared to the matched general population (IRR=141, 95% CI 28.4-2559). African-born women with CHB demonstrated 7.4-times the risk compared to matched African-born peers without CHB (IRR=7.4, 95% CI 1.8-49.8) and 17.7-times the risk compared to matched women from the general population (IRR=17.7, 95% CI 5.3-67.4). Results not shown in tables.

**Discussion**

This study of 3,865 African-born individuals with CHB and no cirrhosis at baseline using prospectively collected nationwide data showed that the IR of HCC exceeds the currently used threshold of 0.2% per year at 54 years in men and 59 years in women, using our statistical model. The cut-off age for which IR exceeds 0.2% per year was though younger if HCV or HDV co-infection with IR exceeding 0.2% at the age group of 20-30 years in men.

The decision from which age to start HCC surveillance in African-born persons, especially for men, is a clinical challenge, with diverging age and sex-specific international recommendations [10,14]. There is a paucity in the literature about the IR data of HCC per age and sex in African-born individuals with non-cirrhotic CHB, in contrast to Asian or Caucasian populations, which could have contributed to the discordant guidelines [21]. Moreover, few longitudinal studies have investigated the incident risk of HCC in African immigrants with CHB [22]. Our study with estimates of sex and age-specific IR can therefore provide some evidence for making clinical recommendations for HCC surveillance in this population. Given the occurring development of HCC in young men (even if low IR) with poor survival at late HCC diagnosis, in persons who would otherwise have gained many years of life expectancy if early HCC diagnosis with curative treatment, it is questionable whether the currently used cost-effective threshold of IR 0.2% is appropriate for this group. To our knowledge, there are no published data describing the cost-effectiveness analyses behind this threshold, despite current usage for recommendations in international guidelines [10]. We have in this study described the age and sex-specific IR, but it is nevertheless difficult to draw any firm conclusion from our model about the optimal age to start HCC surveillance in African men with CHB and no cirrhosis. The results need to be confirmed in further studies, and new cost-effectiveness analyses are probably needed for updated recommendations of HCC surveillance in African-born men.

Several studies in and outside the African continent pointed to the young age and advanced disease stage at HCC diagnosis in the population with African descent, regardless of CHB diagnosis [5]. In a US-based study, African Americans tended to be younger, with more frequent HIV co-infections, and at an advanced stage compared to Caucasians at HCC diagnosis [23]. In a large US Veteran’s Administration (VA) cohort study, CHB patients with HCC and no cirrhosis were more likely to be non-white (African American or Asian), had a family history of HCC and hypertension compared to those with cirrhosis and HCC [24]. US-born African Americans with CHB tended to be older age, have a more frequent history of sexually transmitted diseases, illicit use of drugs and tattoos than foreign-born African Americans with CHB [25]. It is plausible that this young age at HCC diagnosis reflects partially the younger age distribution in migrated African-born population. Genetic susceptibility, early HBV infection, other co-infections, and environmental factors such as aflatoxin B1 have been associated with HCC in the African population [26,27]. In this study, there was a significant difference in the age at immigration to Sweden (as indicator for exposure time in Africa) in persons with or without HCC in the whole population, but no difference was seen in men with or without HCC. According to a forecasting analysis, Hispanics and African Americans are predicted to have the highest HCC incidence rates, especially in age group 35–49-year-old among African American by 2030 in California [28]. The low incidence of cirrhosis or HCC in our cohort is in line with results from a younger Western-African cohort with CHB and a recent register-based Danish study of persons with CHB of any origin, while in contrast to higher rates present in US studies including African American or Black population with CHB [29,30,31]. The low incidences in our cohort could possibly be due the young population, healthy migrant effect, free health care in Sweden with good coverage of antiviral treatment to prevent progress of liver fibrosis in the CHB population, or underestimation of these events using ICD codes in the registers. Also, unrecognized cirrhosis may be present in our cohort, as a recent Swedish analysis has shown that such is frequent at the time of HCC diagnosis in persons with viral hepatitis [32].

In Europe, African migrants, especially from Sub-Saharan Africa, harbor the highest prevalence of CHB [32]. The African population constitutes 15%-45% of the published European cohorts of persons with CHB [9,33,34], but the association of African origin with HCC has not been explored [35,36]. The poor survival in men with HCC in our study indicates a rather late diagnosis of HCC at an advanced stage, which is in line with the description of advanced disease stage at HCC diagnosis in Africans, especially for men from Sub-Saharan Africa [23,37]. Men had more prevalent co-morbidities than women in our study. Diabetes mellitus was not associated with an increased risk of HCC in our cohort in contrast to finding of other studies [38], which might be due to the few HCC outcomes limiting the power to detect such an association. Co-infections with HBV/HCV, HIV, and HDV have been shown to have a synergistic effect on HCC risk and are historically more prevalent in these populations with possible late linkage to care, suboptimal treatment uptake, and adherence [39]. It is unclear if there was any difference between men and women regarding performed HCC surveillance in our cohort, or if certain subgroups had a higher risk of missed cirrhosis diagnosis. In a retrospective analysis of patients with heterogenous HCC etiologies, African Americans had 2-times higher odds of unrecognized cirrhosis, and less likelihood to undergo HCC surveillance compared to Caucasian peers, agreeing with findings from a contemporary French case series [24,40]. In the latter study, where most participants (60%) were from Africa, 14% of HBV-related HCC developed in non-cirrhotic liver, with significantly lower age among patients without cirrhosis compared to those with cirrhosis (51 vs 58 years, respectively) [40].

In our study, 51% of persons with HCC did not have cirrhosis diagnosis at the time of HCC diagnosis. Owing to the oncogenic potential of HBV, ~20-30% of HCC can develop in non-cirrhotic livers [21]. 49% of persons with HCC had received cirrhosis diagnosis prior to HCC diagnosis. However, in 60% of these, the cirrhosis diagnosis was received rather late, with ≤ 6 months prior to HCC diagnosis, possibly reflecting the difficulty to diagnose cirrhosis timely, especially in young men. Poor survival after HCC diagnosis with a median survival of 8.2 months may indicate that these men have not been subject to HCC surveillance prior to HCC diagnoses, with HCC being diagnosed at late stages. The late diagnosis of HCC reflects probably the difficulty to accurately predict the HCC risk in this young African population with no cirrhosis using risk-based HCC surveillance, nevertheless the current Swedish guidelines recommend surveillance from age 40 in men. Despite the free health care in Sweden, we cannot though rule out any disparity in health care due to language barriers or limited socioeconomics, which has been shown in other studies with correlation to poor survival after HCC diagnosis [23].

Our findings highlight the importance of early identification of persons at increased risk of fibrosis progression for timely treatment.

In our model, we demonstrated an incidence rate > 0.2%/year at younger ages when present HCV or HDV co-infection. The HCC cases were however few, resulting in a wide CI for the effect size. The elevated HCC IR in those with co-infections in our study is in line with prior estimates of persons with CHB of diverse origins and thus not only in those of African origin [36].

Our study has some limitations. The few outcomes (only 31 cases of HCC) have limited the statistical power in our analyses, especially on a subgroup level. This despite being a rather large cohort, consisting of 3,865 African-born persons without cirrhosis at baseline, with a long follow-up time of median 11.1 years. The low HCC incidence rate among CHB persons without cirrhosis, leading to few HCC outcomes has been also apparent in other large cohorts [24]. The number of persons with co-infections were few in this cohort, which limits the study to draw any firm conclusion about the effect of co-infections on the risk of HCC. The subgroups from the different African regions were not evenly distributed, with the majority from Eastern Africa and limited sample size from some other regions. More studies are needed to study the risk in people from different parts in Africa. We cannot rule out remaining unaccounted confounders. Related to the nature of this population-based register analysis, we did not assess the individual viral and host risk factors that might affect HCC development. Nevertheless, the effect of NA on the risk of HCC was not assessed. Individuals with more advanced liver disease, who developed cirrhosis, with family history of liver cancer and other risk factors are prescribed NA and an unmatched analysis not considering these confounders might produce biased estimates. However, the international recommendations include only age, sex, and ethnicity as decision parameters to start HCC surveillance in clinical practice. Thus, we could in the context of these parameters explore the relevance of cut-off ages mentioned in the current guidelines of HCC surveillance. It is plausible that we might have underestimated the prevalence of cirrhosis at index date by the usage of ICD codes for cirrhosis diagnosis, thus overestimating the risk of HCC in our cohort with non-cirrhotic CHB patients. Even with this potential overestimation, the IR for HCC exceeds 0.2%/year for men at > 50 years of age, with a substantially higher cut-off age than the recommended starting age for HCC surveillance in 20 or 40 years for African men in some international guidelines [11,12]. We have assumed the cost-effective threshold IR to be 0.2%/year for persons with non-cirrhotic CHB, as used for HCC surveillance in AASLD or EASL guidelines [10,14]. We acknowledge though the need for contemporary cost-effectiveness studies and better risk stratification to support this cut-off data rather than “one-size fits all” approach [42].

The strengths of our study include the nationwide population-based design and usage of prospectively collected data, with the limitation of selection bias. Also, the above-mentioned large sample size and long follow-up time allowed estimation of age and sex-specific IR in our model, in contrast to previously published studies of CHB patients with different origins or smaller cohort studies [43]. While some previous studies have consisted mostly of men, we have data of both sexes with generalizability to both populations [5,24]. Our findings may be generalized to other low-endemic settings, although noting the heterogeneity of African populations living with CHB after migration to the West. To limit surveillance bias, we started the follow-up 6 months after the index date and excluded all deaths, liver cancer, and liver transplantation.

In conclusion, our model indicates that the annual HCC risk of 0.2% is exceeded between age 50-60 years in African-born individuals with non-cirrhotic CHB in Sweden, and with an earlier age if co-infection with HCV or HDV is present. The risk for HCC was not negligible in young men, with poor survival in those who developed HCC. There is however a need to further explore these risks and risk factors for HCC in young African men with CHB, to be able to recommend the starting age for HCC surveillance in this population. There is also a need for new cost-effectiveness analyses specific to this young population at risk for HCC, to be able to provide personalized and cost-effective surveillance.

**References:**

[1] Sung H, Ferlay J, Siegel RL, Laversanne M, Soerjomataram I, Jemal A, et al. Global Cancer Statistics 2020: GLOBOCAN Estimates of Incidence and Mortality Worldwide for 36 Cancers in 185 Countries. CA Cancer J Clin 2021;71:209–49. https://doi.org/10.3322/caac.21660.

[2] World Migration Report 2022 n.d. https://publications.iom.int/system/files/pdf/WMR-2022_0.pdf (accessed February 10, 2023).

[3] Ahmad AA, Falla AM, Duffell E, Noori T, Bechini A, Reintjes R, et al. Estimating the scale of chronic hepatitis B virus infection among migrants in EU/EEA countries. BMC Infect Dis 2018;18:1–14. https://doi.org/10.1186/S12879-017-2921-8/TABLES/5.

[4] Kew MC, Macerollo P. Effect of Age on the Etiologic Role of the Hepatitis B Virus in Hepatocellular Carcinoma in Blacks 1988;94:439–81.

[5] Yang JD, Mohamed EA, Aziz AOA, Shousha HI, Hashem MB, Nabeel MM, et al. Characteristics, management, and outcomes of patients with hepatocellular carcinoma in Africa: a multicountry observational study from the Africa Liver Cancer Consortium. Lancet Gastroenterol Hepatol 2017;2:103–11. https://doi.org/10.1016/S2468-1253(16)30161-3/ATTACHMENT/F39390A3-464F-40A5-895D-929948D88C4A/MMC1.PDF.

[6] Singal AG, Pillai A, Tiro J. Early Detection, Curative Treatment, and Survival Rates for Hepatocellular Carcinoma Surveillance in Patients with Cirrhosis: A Meta-analysis. PLoS Med 2014;11:e1001624. https://doi.org/10.1371/JOURNAL.PMED.1001624.

[7] Bruix J, Sherman M. Management of hepatocellular carcinoma: an update. Hepatology 2011;53:1020–2. https://doi.org/10.1002/HEP.24199.

[8] Sarasin FP, Giostra E, Hadengue A. Cost-effectiveness of screening for detection of small hepatocellular carcinoma in western patients with Child-Pugh class A cirrhosis. Am J Med 1996;101:422–34. https://doi.org/10.1016/S0002-9343(96)00197-0.

[9] Duberg AS, Lybeck C, Fält A, Montgomery S, Aleman S. Chronic hepatitis B virus infection and the risk of hepatocellular carcinoma by age and country of origin in people living in Sweden: A national register study. Hepatol Commun 2022. https://doi.org/10.1002/HEP4.1974.

[10] Terrault NA, Lok ASF, Mcmahon BJ, Chang K-M, Hwang JP, Jonas MM, et al. Update on Prevention, Diagnosis, and Treatment of Chronic Hepatitis B: AASLD 2018 Hepatitis B Guidance American Association for the study of liver diseases. vol. 67. 2018. https://doi.org/10.1002/hep.29800.

[11] Omata M, Cheng AL, Kokudo N, Kudo M, Lee JM, Jia J, et al. Asia–Pacific clinical practice guidelines on the management of hepatocellular carcinoma: a 2017 update. Hepatol Int 2017;11:317–70. https://doi.org/10.1007/s12072-017-9799-9.

[12] Burak KW, Sherman M. Hepatocellular carcinoma: Consensus, controversies and future directions: A report from the Canadian Association for the Study of the Liver Hepatocellular Carcinoma Meeting. Can J Gastroenterol Hepatol 2015;29:178. https://doi.org/10.1155/2015/824263.

[13] Zhou J, Sun HC, Wang Z, Cong WM, Wang JH, Zeng MS, et al. [Guidelines for diagnosis and treatment of primary liver cancer in China (2019 edition)]. Zhonghua Gan Zang Bing Za Zhi 2020;28:235–60. https://doi.org/10.3760/CMA.J.ISSN.1007-3418.2020.02.004.

[14] Llovet JM, Ducreux M, Lencioni R, Di Bisceglie AM, Galle PR, Dufour JF, et al. EASL-EORTC Clinical Practice Guidelines: Management of hepatocellular carcinoma. J Hepatol 2012;56:908–43. https://doi.org/10.1016/j.jhep.2011.12.001.

[15] Papatheodoridis G, Dalekos G, Sypsa V, Yurdaydin C, Buti M, Goulis J, et al. PAGE-B predicts the risk of developing hepatocellular carcinoma in Caucasians with chronic hepatitis B on 5-year antiviral therapy. J Hepatol 2016;64:800–6.

[16] Kim JH, Kim YD, Lee M, Jun BG, Kim TS, Suk KT, et al. Modified PAGE-B score predicts the risk of hepatocellular carcinoma in Asians with chronic hepatitis B on antiviral therapy. J Hepatol 2018;69:1066–73. https://doi.org/10.1016/J.JHEP.2018.07.018.

[17] Screening – övervakning (surveillance) av riskgrupper - RCC Kunskapsbanken n.d. https://kunskapsbanken.cancercentrum.se/diagnoser/levercellscancer/vardprogram/screening--overvakning-surveillance-av-riskgrupper/ (accessed March 12, 2023).

[18] UNSD — Methodology n.d. https://unstats.un.org/unsd/methodology/m49/ (accessed July 26, 2022).

[19] Simon TG, Duberg A-S, Aleman S, Chung RT, Chan AT, Ludvigsson JF. Association of Aspirin with Hepatocellular Carcinoma and Liver-Related Mortality. N Engl J Med 2020;382:1018–28. https://doi.org/10.1056/NEJMOA1912035.

[20] Törner A, Stokkeland K, Svensson Å, Dickman PW, Hultcrantz R, Montgomery S, et al. The underreporting of hepatocellular carcinoma to the cancer register and a log-linear model to estimate a more correct incidence. Hepatology 2017;65:885–92. https://doi.org/10.1002/hep.28775.

[21] Yen YH, Cheng YF, Wang JH, Lin CC, Wang CC. Characteristics and etiologies of hepatocellular carcinoma in patients without cirrhosis: When East meets West. PLoS One 2021;16:e0244939. https://doi.org/10.1371/JOURNAL.PONE.0244939.

[22] Mitchell T, Nayagam JS, Dusheiko G, Agarwal K. Health inequalities in the management of chronic hepatitis B virus infection in patients from sub-Saharan Africa in high-income countries. JHEP Reports 2023;5:100623. https://doi.org/10.1016/j.jhepr.2022.100623.

[23] Estevez J, Yang JD, Leong J, Nguyen P, Giama NH, Zhang N, et al. Clinical Features Associated with Survival Outcome in African-American Patients with Hepatocellular Carcinoma. American Journal of Gastroenterology 2019;114:80–8. https://doi.org/10.1038/S41395-018-0261-Y.

[24] Chayanupatkul M, Omino R, Mittal S, Kramer JR, Richardson P, Thrift AP, et al. Hepatocellular carcinoma in the absence of cirrhosis in patients with chronic hepatitis B virus infection. J Hepatol 2017;66:355–62. https://doi.org/10.1016/J.JHEP.2016.09.013.

[25] Hassan MA, Kim WR, Li R, Smith CI, Fried MW, Sterling RK, et al. Characteristics of US-Born Versus Foreign-Born Americans of African Descent With Chronic Hepatitis B. Am J Epidemiol 2017;186:356. https://doi.org/10.1093/AJE/KWX064.

[26] Khalili M, Lombardero M, Chung RT, Terrault NA, Ghany MG, Kim WR, et al. Diabetes and prediabetes in patients with hepatitis B residing in North America. Hepatology 2015;62:1364–74. https://doi.org/10.1002/HEP.28110/SUPPINFO.

[27] Loomba R, Liu J, Yang HI, Lee MH, Lu SN, Wang LY, et al. Synergistic Effects of Family History of Hepatocellular Carcinoma and Hepatitis B Virus Infection on Risk for Incident Hepatocellular Carcinoma. Clin Gastroenterol Hepatol 2013;11:1636. https://doi.org/10.1016/J.CGH.2013.04.043.

[28] Han SS, Kelly SP, Li Y, Yang B, Nguyen M, So S, et al. Changing Landscape of Liver Cancer in California: A Glimpse Into the Future of Liver Cancer in the United States. JNCI: Journal of the National Cancer Institute 2019;111:550–6. https://doi.org/10.1093/JNCI/DJY180.

[29] Shimakawa Y, Lemoine M, Njai HF, Bottomley C, Ndow G, Goldin RD, et al. Natural history of chronic HBV infection in West Africa: a longitudinal population-based study from The Gambia. Gut 2016;65:2007–16. https://doi.org/10.1136/GUTJNL-2015-309892.

[30] Khalili M, Leonard KR, Ghany MG, Hassan M, Roberts LR, Sterling RK, et al. Racial Disparities in Treatment Initiation and Outcomes of Chronic Hepatitis B Virus Infection in North America. JAMA Netw Open 2023;6:e237018–e237018. https://doi.org/10.1001/JAMANETWORKOPEN.2023.7018.

[31] Bollerup S, Engsig F, Hallager S, Mocroft A, Roege BT, Laursen AL, et al. Incidence of Hepatocellular Carcinoma and Decompensated Liver Cirrhosis and Prognostic Accuracy of the PAGE-B HCC Risk Score in a Low Endemic Hepatitis B Virus Infected Population. J Hepatocell Carcinoma 2022;2022:1093. https://doi.org/10.2147/JHC.S372571.

[32] Ahmad AA, Falla AM, Duffell E, Noori T, Bechini A, Reintjes R, et al. Estimating the scale of chronic hepatitis B virus infection among migrants in EU/EEA countries. BMC Infect Dis 2018;18:1–14. https://doi.org/10.1186/S12879-017-2921-8/TABLES/5.

[33] Bollerup S, Hallager S, Engsig F, Mocroft A, Krarup H, Madsen LG, et al. Mortality and cause of death in persons with chronic hepatitis B virus infection versus healthy persons from the general population in Denmark. J Viral Hepat 2022;29:727–36. https://doi.org/10.1111/JVH.13713.

[34] Chevaliez S, Roudot-Thoraval F, Brouard C, Gordien E, Zoulim F, Brichler S, et al. Clinical and virological features of chronic hepatitis B in the French national surveillance program, 2008–2012: A cross-sectional study. JHEP Reports 2022;4. https://doi.org/10.1016/j.jhepr.2022.100593.

[35] Niederau C, Amani A, Thiel A. Long-term follow-up of HBsAg-positive patients in Germany. Eur J Gastroenterol Hepatol 2016;28:48–56. https://doi.org/10.1097/MEG.0000000000000487.

[36] Mallet V, Hamed K, Schwarzinger M. Prognosis of patients with chronic hepatitis B in France (2008-2013): A nationwide, observational and hospital-based study. vol. 87. n.d.

[37] Yang JD, Gyedu A, Afihene MY, Duduyemi BM, Micah E, Kingham TP, et al. Hepatocellular Carcinoma Occurs at an Earlier Age in Africans, Particularly in Association With Chronic Hepatitis B. Am J Gastroenterol 2015;110:1629–31. https://doi.org/10.1038/AJG.2015.289.

[38] Tan Y, Wei S, Zhang W, Yang J, Yang J, Yan L. Cancer Management and Research ISSN: (Print) (Online) Journal homepage: https://www.tandfonline.com/loi/dcmr20 Type 2 diabetes mellitus increases the risk of hepatocellular carcinoma in subjects with chronic hepatitis B virus infection: a meta-analysis and systematic review Cancer Management and Research Dovepress Type 2 diabetes mellitus increases the risk of hepatocellular carcinoma in subjects with chronic hepatitis B virus infection: a meta-analysis and systematic review. Cancer Manag Res 2019:11–705. https://doi.org/10.2147/CMAR.S188238.

[39] Valerio H, Alavi M, Law M, Tillakeratne S, Amin J, Janjua NZ, et al. High hepatitis C treatment uptake among people with recent drug dependence in New South Wales, Australia. J Hepatol 2021;74:293–302. https://doi.org/10.1016/J.JHEP.2020.08.038.

[40] Allaire M, El Hajj W, Brichler S, Diallo K, Fanica D, Blaise L, et al. Prior surveillance and antiviral treatment improve the prognosis of HCC developed in HBV patients in the West. Clin Res Hepatol Gastroenterol 2021;45. https://doi.org/10.1016/J.CLINRE.2020.03.030.

[41] Henriksson M, Bjornsson B, Eilard S, Lindell M, Stromberg G, Björnsson B, et al. Treatment patterns and survival in patients with hepatocellular carcinoma in the Swedish national registry SweLiv 2019. https://doi.org/10.1002/bjs5.50226.

[42] EASL Policy Statement Risk-based surveillance for hepatocellular carcinoma among patients with cirrhosis POLICY STATEMENT n.d.

[43] Udompap P, Moscoso CG, Anugwom C, Kc M, Lim N, Lake J, et al. Viral Hepatitis Among African Immigrants with Hepatocellular Carcinoma in Minnesota: High Prevalence Yet Low Awareness. J Immigr Minor Health 2022. https://doi.org/10.1007/S10903-022-01400-1.

**Main Tables:**

- Table 1. Baseline characteristics of African-born persons with chronic hepatitis B (CHB) and without liver cirrhosis, living in Sweden. The characteristics for all and grouped by sex are shown.
- Table 2: Incidence rate ratios (IRR) for HCC derived from Poisson regression models presenting the association of variables in a chronic hepatitis B cohort of persons with African origin.

**Supplementary Tables**

- Table S1: Baseline characteristics of African-born individuals with CHB without liver cirrhosis per African region of birth.
- Table S2. Baseline characteristics of African-born men with CHB, and in comparator cohorts from the same area of origin and the general population without HBV infection.
- Table S3: Characteristics and survival of African-born persons with CHB who developed hepatocellular carcinoma, by sex.
- Table S4: Characteristics and survival of persons with CHB who developed HCC per African region of birth.

| ***Table 1.* *Baseline characteristics of African-born persons with chronic hepatitis B (CHB) and without liver cirrhosis, living in Sweden. The characteristics for all and grouped by sex are shown, with P-value for comparison between men and women.*** | | | | |
| --- | --- | --- | --- | --- |
| **Characteristics** | **All (n, %)** | **Men (n, %)** | **Women (n, %)** | **P-value** |
| **Total** | 3865 | 2266 (58.6) | 1599 (41.4) | <0.001 |
| **Age at immigration, mean (SD), years^¶^** | 27.5 (11.6) | 27.5 (11.5) | 27.6 (11.9) | 0.89 |
| **Age at start of follow-up, mean (SD), years** | 32.1 (11.2) | 32.5 (11.2) | 31.4 (11.2) | <0.001 |
| **Age groups at start of follow-up, years** |  |  |  | <0.001 |
| 18–29 | 1805 (46.7) | 994 (43.9) | 811 (50.7) |  |
| 30–39 | 1252 (32.4) | 715 (31.6) | 537 (33.6) |  |
| 40–49 | 531 (13.7) | 398 (17.6) | 133 (8.3) |  |
| >50 | 277 (7.2) | 159 (7.0) | 118 (7.4) |  |
| **HBV diagnosis date, years** | | | | <0.001 |
| 1990–1999 | 861 (22.3) | 469 (20.7) | 392 (24.5) |  |
| 2000–2009 | 1554 (40.2) | 895 (39.5) | 659 (41.2) |  |
| 2010–2015 | 1450 (37.5) | 902 (39.8) | 548 (34.3) |  |
| **Education level (years in school), years** | | | | <0.001 |
| ≤9 | 1395 (36.1) | 778 (34.3) | 617 (38.6) |  |
| 10–12 | 1246 (32.2) | 756 (33.4) | 490 (30.6) |  |
| >13 | 858 (22.2) | 567 (25.0) | 291 (18.2) |  |
| Missing | 366 (9.5) | 165 (7.3) | 201 (12.6) |  |
| **African region of birth** | | | | 0.90 |
| Northern | 261 (6.8) | 152 (6.7) | 109 (6.8) |  |
| Eastern | 2478 (64.1) | 1455 (64.2) | 1023 (64.0) |  |
| Middle | 240 (6.2) | 152 (6.7) | 94 (5.9) |  |
| Western | 874 (22.6) | 505 (22.3) | 369 (23.1) |  |
| Southern | 12 (0.3) | 8 (0.4) | 4 (0.3) |  |
| **Co-morbidities** | | | |  |
| HCV co-infection | 194 (5.0) | 130 (5.7) | 64 (4.0) | 0.02 |
| HDV co-infection | 105 (2.7) | 63 (2.8) | 42 (2.6) | 0.77 |
| HIV co-infection | 126 (3.3) | 75 (3.3) | 51 (3.2) | 0.84 |
| Alcohol overconsumption | 93 (2.4) | 75 (3.3) | 18 (1.1) | <0.001 |
| Diabetes mellitus | 326 (8.4) | 221 (9.8) | 105 (6.6) | <0.001 |
| Obesity | 28 (0.7) | 3 (0.1) | 25 (1.6) | <0.001 |
| Drug misuse | 60 (1.6) | 47 (2.1) | 13 (0.8) | 0.002 |
| **Co-medications** | | | |  |
| Interferon therapy | 39 (1.0) | 32 (1.4) | 7 (0.4) | 0.003 |
| Nucleos(t)ide analogues | 260 (6.7) | 178 (7.9) | 82 (5.1) | <0.001 |
| Statin | 224 (5.8) | 157 (6.9) | 67 (4.2) | <0.001 |
| Aspirin | 135 (3.5) | 81 (3.6) | 54 (3.4) | 0.74 |
| **Follow-up time, years** | | | |  |
| Mean (SD) | 12.4 (6.7) | 12.0 (6.6) | 13.1 (6.8) | <.0001 |
| *Abbreviations: CHB=chronic HBV; SD= standard deviation; HCV=hepatitis C virus; HDV=hepatitis Delta virus;* **^¶^***=The age or year at first immigration event, if >one event of immigration; *=HIV and or HCV and/or HDV and/or DM.* | | | | |

| **Table 2: Incidence rate ratios (IRR) for HCC derived from Poisson regression models presenting the association of variables in a chronic hepatitis B cohort of persons with African origin.** | | | | | | | | | |
| --- | --- | --- | --- | --- | --- | --- | --- | --- | --- |
|  | **Univariable** | | | **Base model (multivariable)** | | | **Adjusted estimates^#^** | | |
| **Predictors** | **IRR** | **95% CI** | **p** | **IRR** | **95% CI** | **p** | **IRR** | **95% CI** | **p** |
| **Base Model** | | | | | | | | | |
| Age (centered) | 1.07 | 1.04-1.09 | 0* | 1.12 | 1.07-1.18 | 0* | - | - | - |
| Men | 2.66 | 1.21-6.68 | 0.02* | 7.13 | 2.08-42.31 | 0.01* | - | - | - |
| Age * men | - | - | - | 0.94 | 0.88-0.99 | 0.03* | - | - | - |
| **Co-morbidities** | | | | | | | | | |
| HCV co-infection | 4 | 1.49-9.14 | 0.002* | - | - | - | **2.75** | **1.01-6.37** | **0.03*** |
| HDV co-infection | 4.27 | 1.02-12 | 0.02* | - | - | - | **4.47** | **1.06-12.84** | **0.02*** |
| HIV co-infection | 1.99 | 0.32-6.6 | 0.3 | - | - | - | 2.01 | 0.32-6.73 | 0.3 |
| Diabetes mellitus | 1.81 | 0.61-4.33 | 0.2 | - | - | - | 0.97 | 0.32-2.43 | >0.9 |
| **African region of origin (vs Eastern)** | | | | | | | | | |
| Middle | 2.73 | 0.79-7.39 | 0.07* | - | - | - | **3.82** | **1.08-10.65** | **0.02*** |
| Northern | 1.6 | 0.37-4.76 | 0.5 | - | - | - | 1.73 | 0.4-5.3 | 0.4 |
| Western | 1.15 | 0.44-2.66 | 0.8 | - | - | - | 1.77 | 0.66-4.32 | 0.2 |
| *CI= Confidence Intervals; p=p-value.* **^#^***Adjusted estimates are univariable estimates adjusted for the Base Model.* **P-values < 0.05.* | | | | | | | | | |

| ***Table S1: Baseline characteristics of African-born individuals with chronic hepatitis B without cirrhosis per African region of birth*** | | | | | | |
| --- | --- | --- | --- | --- | --- | --- |
|  | **Middle** | **Eastern** | **Northern** | **Southern** | **Western** | **P-value** |
| **Total, n (%)** | 240 | 2478 | 261 | 12 | 874 | <0.001 |
| Men, n (%) | 146 (60.8) | 1455 (58.7) | 152 (58.2) | 8 (66.7) | 505 (57.8) | 0.15 |
| **Age at start of follow-up** | | | | | |  |
| All, mean (SD) | 29.8 (9.1) | 32.7 (12.0) | 35.3 (9.5) | 32.0 (11.1) | 29.9 (9.6) | <0.001 |
| Men, mean (SD), years | 30.2 (8.8) | 32.8 (11.7) | 36.9 (9.7) | 34.9 (12.1) | 31.0 (10.5) | <0.001 |
| Women, mean (SD), years | 29.4 (9.4) | 32.6 (12.3) | 33.0 (8.8) | 26.1 (6.1) | 28.3 (8.0) |  |
| **Age groups (years), n (%)** | | | | | | <0.001 |
| 20–29 | 125 (52.1) | 1139 (46.0) | 78 (29.9) | 6 (50.0) | 457 (52.3) |  |
| 30–39 | 90 (37.5) | 759 (30.6) | 113 (43.3) | 3 (25.0) | 287 (32.8) |  |
| 40–49 | 16 (6.7) | 359 (14.5) | 46 (17.6) | 3 (25.0) | 107 (12.2) |  |
| ≥50 | 9 (3.8) | 221 (8.9) | 24 (9.2) | 0 | 23 (2.6) |  |
| **HBV diagnosis date/date of matching, n (%)** | | | | | | <0.001 |
| 1990–1999 | 24 (10.0) | 594 (24.0) | 65 (24.9) | 1 (8.3) | 177 (20.3 |  |
| 2000–2009 | 131 (54.6) | 907 (36.46) | 92 (35.2) | 7 (58.3) | 417 (47.7) |  |
| 2010–2015 | 85 (35.4) | 977 (39.4) | 104 (39.8) | 4 (33.3) | 280 (32.0) |  |
| **Education level (years in school), n (%)** | | | | | | <0.001 |
| = <9 | 41 (17.1) | 1056 (42.6) | 68 (26.1) | 1 (8.3) | 229 (26.2) |  |
| 10–12 | 71 (29.6) | 718 (29.0) | 101 (38.7) | 5 (41.7) | 351 (40.2) |  |
| >=13 | 114 (47.5) | 410 (16.5) | 79 (30.3) | 5 (41.7) | 250 (28.6) |  |
| Unknown | 14 (5.8) | 294 (11.9) | 13 (5.0) | 1 (8.3) | 44 (5.0) |  |
| **Co-morbidities, n (%)** | | | | | |  |
| HCV | 19 (7.9) | 113 (4.6) | 19 (7.3) | 1 (8.3) | 42 (4.8) | 0.08 |
| HDV | 6 (2.5) | 61 (2.5) | 10 (3.8) | 0 | 28 (3.2) | 0.53 |
| HIV | 10 (4.2) | 71 (2.9) | 3 (1.1) | 5 (41.7) | 37 (4.2) | <0.001 |
| Alcohol overconsumption | 2 (0.8) | 54 (2.2) | 11 (4.2) | 1 (8.3) | 25 (2.9) | 0.06 |
| DM | 15 (6.3) | 225 (9.1) | 36 (13.8) | 0 | 50 (5.7) | <0.001 |
| Drug misuse | 0 | 29 (1.2) | 11 (4.2) | 0 | 20 (2.3) | <0.001 |
| **Therapies, n (%)** | | | | | |  |
| Interferon | 0 | 20 (0.8) | 4 (1.5) | 0 | 15 (1.7) | 0.07 |
| Nucleos(t)ide analogues | 25 (10.4) | 130 (5.2) | 29 (11.1) | 1 (8.3) | 75 (8.6) | <0.001 |
| Aspirin | 8 (3.3) | 70 (2.8) | 17 (6.5) | 1 (8.3) | 39 (4.5) | 0.02 |
| Statin | 10 (4.2) | 146 (5.9) | 29 (11.1) | 0 | 39 (4.5) | 0.001 |
| **Follow-up time in years** | | | | | |  |
| Mean (SD) | 11.1 (5.5) | 12.4 (6.9) | 13.1 (5.3) | 10.4 (5.3) | 12.7 (6.2) | 0.02 |
| *Abbreviations: CHB=chronic hepatitis B; SD= standard deviation; IQR=interquartile range; HCV=hepatitis C virus; HDV=hepatitis Delta virus; HIV=human immunodeficiency virus;DM=diabetes mellitus. Numbers are presented as frequencies and % percentages within column)* | | | | | | |

| ***Table S2.* *Baseline characteristics of African-born men with CHB, and in comparator cohorts from the same area of origin and the general population without HBV infection.*** | | | | |
| --- | --- | --- | --- | --- |
| **Characteristics** | **Men with CHB (n, %)** | **Cohort from the same area of origin (n, %)** | **Cohort from the general population (n, %)** | **P-value** |
| **Total*** | 2266 (58.6) | 5048 (59.5) | 23,188 (59.0) |  |
| **Age at start of follow-up (years)** | | | |  |
| All, mean (SD) | 32.5 (11.2) | 32.9 (10.9) | 32.9 (11.4) | 0.16 |
| **Age groups, n (%)** |  |  |  | 0.20 |
| 18–29 | 994 (43.9) | 2116 (41.9) | 9877 (42.6) |  |
| 30–39 | 715 (31.6) | 1652 (32.7) | 7306 (31.5) |  |
| 40–49 | 398 (17.6) | 914 (18.1) | 4149 (17.9) |  |
| >50 | 159 (7.0) | 366 (7.3) | 1856 (8.0) |  |
| **HBV diagnosis date (years), n (%)/matching date for comparators** | | | | <0.001 |
| 1990–1999 | 469 (20.7) | 1034 (20.5) | 4762 (20.5) |  |
| 2000–2009 | 895 (39.5) | 1789 (35.4) | 9176 (39.6) |  |
| 2010–2015 | 902 (39.8) | 2225 (44.1) | 9250 (39.9) |  |
| **Education level (years in school), n (%)** | | | | <0.001 |
| ≤9 | 778 (34.3) | 1613 (32.0) | 4095 (17.7) |  |
| 10–12 | 756 (33.4) | 1882 (37.3) | 10,109 (43.6) |  |
| >13 | 567 (25.0) | 1186 (23.5) | 7699 (33.2) |  |
| Missing | 165 (7.3) | 367 (7.3) | 1285 (5.5) |  |
| **African region of birth, n (%)** | | | | 0.01 |
| Northern | 152 (6.7) | 346 (6.9) | 185 (0.8) |  |
| Eastern | 1455 (64.2) | 3643 (72.2) | 862 (3.7) |  |
| Middle | 146 (6.4) | 202 (4.0) | 42 (0.2) |  |
| Western | 505 (22.3) | 847 (16.8) | 93 (0.4) |  |
| Southern | 8 (0.4) | 8 (0.2) | 6 (0.03) |  |
| Other | - | 2 (0.0) | 22,000 (94.9) |  |
| **Co-morbidities, n (%)** | | | |  |
| HCV co-infection | 130 (5.7) | 43 (0.9) | 11 (0.05) | <0.001 |
| HDV co-infection | 63 (2.8) | - | - |  |
| HIV co-infection | 75 (3.3) | 45 (0.9) | 31 (0.1) | <0.001 |
| Alcohol overconsumption | 75 (3.3) | 130 (2.6) | 746 (3.2) | 0.05 |
| DM | 221 (9.8) | 477 (9.4) | 1143 (4.9) | <0.001 |
| Drug misuse | 47 (2.1) | 95 (1.9) | 226 (1.0) | <0.001 |
| **Co-medications, n (%)** | | | |  |
| Interferon therapy | 32 (1.4) | 8 (0.2) | 5 (0.02) | <0.001 |
| Nucleos(t)ide analogues | 178 (7.9) | 8 (0.2) | 5 (0.02) | <0.001 |
| Statin | 157 (6.9) | 417 (8.3) | 2025 (8.7) | 0.01 |
| Aspirin | 81 (3.6) | 185 (3.7) | 1075 (4.6) | 0.001 |
| **Follow-up time (years)** | | | |  |
| Mean (SD) | 11.9 (6.6) | 11.8 (6.7) | 12.0 (6.6) | 0.04 |
| *Abbreviations: *Compared to the total respective cohort; CHB=chronic HBV; SD= standard deviation; HCV=hepatitis C virus; HDV=hepatitis Delta; HIV=human immunodeficiency virus; Diabetes mellitus. ^=HIV and or HCV and/or HDV and/or DM. Numbers are rounded to the 2^nd^ decimal. Frequencies are presented as numbers and percentages within column.* | | | | |
|  | | | | |

| ***Table S3: Characteristics and survival of African-born persons with chronic hepatitis B with hepatocellular carcinoma, by sex.*** | | | | |
| --- | --- | --- | --- | --- |
|  | **All** | **Men** | **Women** | **P-value** |
| Total, n (%) by row | 31 (100) | 24 (77.4) | 7 (22.6) | <0.001 |
| **Age at immigration, mean (SD), years** | 35.5 (16.4) | 30.4 (12.1) | 53.2 (18.0) | 0.22 |
| **Age at HCC diagnosis, mean (SD), years** | 51.4 (16.6) | 46.8 (14.7) | 67.0 (13.7) | 0.003 |
| **Date of HBV diagnosis, n (%)** | | | | 0.37 |
| 1990–1999 | 10 (32.3) | 8 (33.3) | 2 (28.6) |  |
| 2000–2009 | 17 (54.8) | 14 (58.3) | 3 (42.9) |  |
| 2010–2015 | 4 (12.9) | 2 (8.3) | 2 (28.6) |  |
| **Level of education, n (%), years in school** | | | | 0.02 |
| ≤9 | 4 (12.9) | 2 (8.3) | 2 (28.6) |  |
| 10–12 | 12 (38.7) | 11 (45.8) | 1 (14.3) |  |
| >13 | 8 (26.8) | 8 (33.3) | 0 |  |
| Missing | 7 (22.6) | 3 (12.5) | 4 (57.1) |  |
| **African region of birth, n (%)** | | | | 0.21 |
| Northern | 3 (12.9) | 3 (12.5) | 0 |  |
| Eastern | 17 (54.8) | 11 (45.8) | 6 (85.7) |  |
| Middle | 4 (12.9) | 3 (11.1) | 1 (14.3) |  |
| Western | 7 (22.6) | 7 (29.2) | 0 |  |
| **Comorbidities, n (%)** | | | |  |
| HCV | 6 (19.4) | 4 (16.7) | 2 (28.6) | 0.49 |
| HDV | 3 (9.7) | 3 (12.5) | 0 | 0.20 |
| HIV | 2 (6.5) | 2 (8.3) | 0 | 0.30 |
| DM | 5 (16.1) | 5 (20.8) | 0 | 0.56 |
| Cirrhosis diagnosis during follow-up | 15 (48.4) | 11 (45.8) | 4 (57.1) | 0.59 |
| Received IFN | 2 (6.5) | 2 (8.3) | 0 | 0.43 |
| Received nucleos(t)ide analogues | 15 (48.4) | 11 (45.8) | 4 (57.1) | 0.59 |
| Received liver transplant after HCC diagnosis | 2 (6.5) | 1 (4.2) | 1 (14.3) | 0.33 |
| **Survival excluding those diagnosed from Death Register (n=13)** | | | | |
| Survival at 1 year, n (%) | 8 (25.8) | 5 (20.8) | 3 (42.9) | 0.01 |
| Survival time, median (IQR), months | 8.2 (2.9-89.2) | 6.7 (2.0-82.3) | 64.4 (17.1-104.1) | 0.29 |
| **Follow-up time in years** | | | | |
| Median (IQR) | 9.2 (3.9–14.3) | 8.5 (3.5–13.7) | 9.2 (6.0–15.8) | 0.97 |
| *Abbreviations: n= number; HCC= hepatocellular carcinoma; SD= standard deviation; IQR=interquartile range; HCV=hepatitis C virus; HDV=hepatitis Delta virus; HIV=human immunodeficiency virus; DM=diabetes mellitus;IFN=interferon; na=not applicable. Numbers are rounded to the 2nd decimal. Proportions are presented by column. NB: none of persons with CHB who developed HCC had a record of alcohol overconsumption or obesity.* | | | | |

| ***Table S4: Characteristics and survival of persons with chronic hepatitis B with HCC per African region of birth*** | | | | | |
| --- | --- | --- | --- | --- | --- |
|  | **Middle** | **Eastern** | **Western** | **Northern** | **P-value** |
| **Total, n (%)** | 240 (6.2) | 2478 (64.1) | 874 (22.6) | 261 (6.8) | <0.001 |
| Number of persons with HCC, n (%) | 4 (1.7) | 17 (0.7) | 7 (0.8) | 3 (1.2) | 0.03 |
| Men, n (%) | 3 (75) | 11 (64.7) | 7 (100) | 3 (100) |  |
| Women, n (%) | 1 (25) | 6 (35.3) | 0 | 0 |  |
| **Age at HCC diagnosis (years)** |  | | | |  |
| All, median (IQR) | 36.9 (28.7–69.3) | 49.5 (38.0–67.6) | 54.9 (35.0–62.9) | 59.7 (36.8–59.7) | 0.64 |
| Men, mean (SD) | 34.1 (9.2) | 43.9 (12.8) | 51.9 (13.3) | 58.5 (21.2) | (0.05) ** |
| Women, mean (SD) | 77.5 | 65.2 (14.1) | 0 | 0 | 0.57 |
| **Date of HBV diagnosis, n (%)** | | |  |  | 0.51 |
| 1990–1999 | 0 | 6 (35.3) | 2 (28.6) | 2 (66.7) |  |
| 2000–2009 | 3 (75.0) | 9 (52.9) | 4 (57.1) | 1 (33.3) |  |
| 2010–2015 | 1 (25.0) | 2 (11.8) | 1 (14.3) | 0 |  |
| **Level of education (years in school), n (%)** | | | | | 0.70 |
| ≤ 9 | 1 (25) | 3 (17.6) | 0 | 0 |  |
| 10–12 | 1 (25) | 5 (29.4) | 5 (71.4) | 1 (33.3) |  |
| > 13 | 1 (25) | 5 (29.4) | 1 (14.3) | 1 (33.3) |  |
| Missing | 1 (25) | 4 (23.5) | 1 (14.3) | 1 (33.3) |  |
| **Co-morbidities, n (%)** |  | | | |  |
| HCV | 0 | 5 (29.4) | 0 | 1 (33.3) | 0.11 |
| HDV | 1 (25.0) | 1 (5.9) | 0 | 1 (33.3) | 0.28 |
| HIV | 0 | 1 (5.9) | 1 (14.3) | 0 | 0.68 |
| DM | 1 (25.0) | 1 (5.9) | 1 (14.3) | 2 (66.7) | 0.13 |
| Cirrhosis diagnosis during follow-up | 3 (75.0) | 8 (47.1) | 1 (14.3) | 3 (100) | 0.03 |
| Received IFN therapy | 0 | 1 (5.9) | 1 (14.3) | 0 | 0.75 |
| Received nucleos(t)ide analogues | 3 (75.0) | 7 (41.2) | 3 (42.9) | 2 (66.7) | 0.57 |
| Received liver transplant after HCC diagnosis | 0 | 2 (11.7) | 0 | 0 |  |
| **Survival excluding those diagnosed from Death Register (n=13), n (%)** | | | | | |
| Survival at 1 year | 0 | 7 (41.2) | 1 (14.3) | 0 | 0.42 |
| Survival time^&^, median (IQR), months | 0.0 | 7.7 (0.0-81.6) | 0.5 (0.0-6.5) | 0.5 (0.0-0.5) | 0.01 |
| **Follow-up time in years** |  | | | |  |
| Median (IQ) | 3.01 (1.2–4.4) | 9.4 (5.6–11.7) | 10.0 (4.8–18.7) | 14.3 (3.9–14.3) | 0.002 |
| *Abbreviations: n=number; HCC= hepatocellular carcinoma; SD= standard deviation; IQR=interquartile range; HCV=hepatitis C virus; HDV=hepatitis Delta virus; HIV=human immunodeficiency virus; DM=diabetes mellitus;na=not applicable. * Including patients who underwent liver transplantation. NB: none of the persons with CHB who developed HCC had a record of alcohol overconsumption or obesity. **Middle vs Northern Africa was significant younger (p=0.04), Middle vs Western Africa was marginally significant at (p=0.05)* | | | | | |

**Main Figures:**

- Figure 1: Flow-chart of the study population
- Figure 2: Box plot showing age at HCC diagnosis in African-born persons with chronic hepatitis B (CHB) and without cirrhosis, by sex and region of birth.
- Figure 3: The incidence rate of HCC per 100 person-year in African-born persons with chronic hepatitis B (CHB) and without cirrhosis, by sex.

**Supplementary Figures:**

- Figure S1: The incidence rates of hepatocellular carcinoma (HCC) per 100 person-year with 95% confidence intervals in African-born persons with chronic hepatitis B (CHB) and without cirrhosis, in persons with HCV or HDV co-infection (Figure a), and per African region of birth (Figure b).
- Figure S2: The incidence rate of HCC in African-born men and women with chronic hepatitis B (CHB), and in matched comparators from the same country of origin and the general population. Y-axis represents IR/100PYs. X-axis represents the age at baseline.


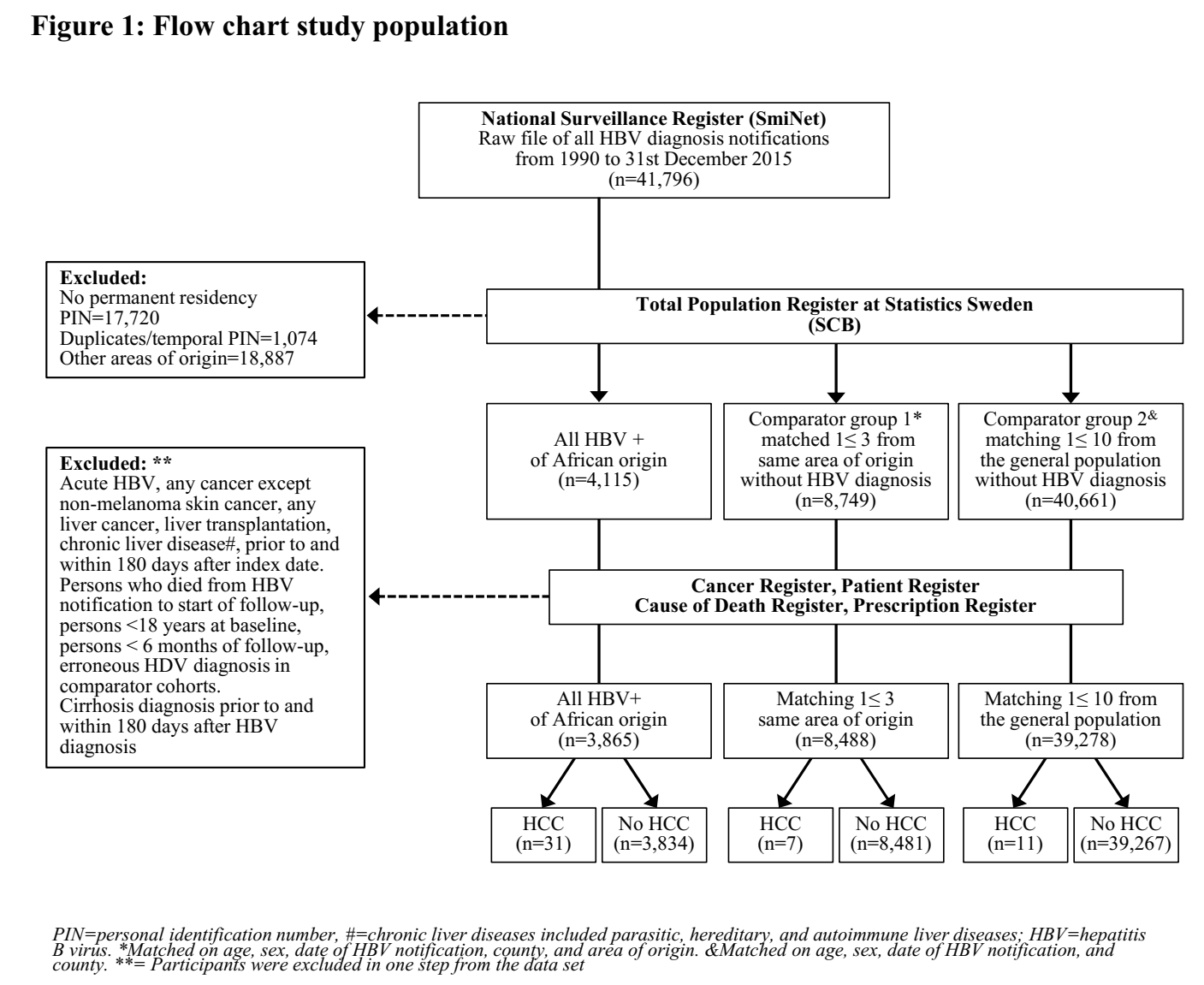


**Figure 2: Box plot showing age at HCC diagnosis in African-born persons with chronic hepatitis B (CHB) and without cirrhosis, by sex and region of birth.**


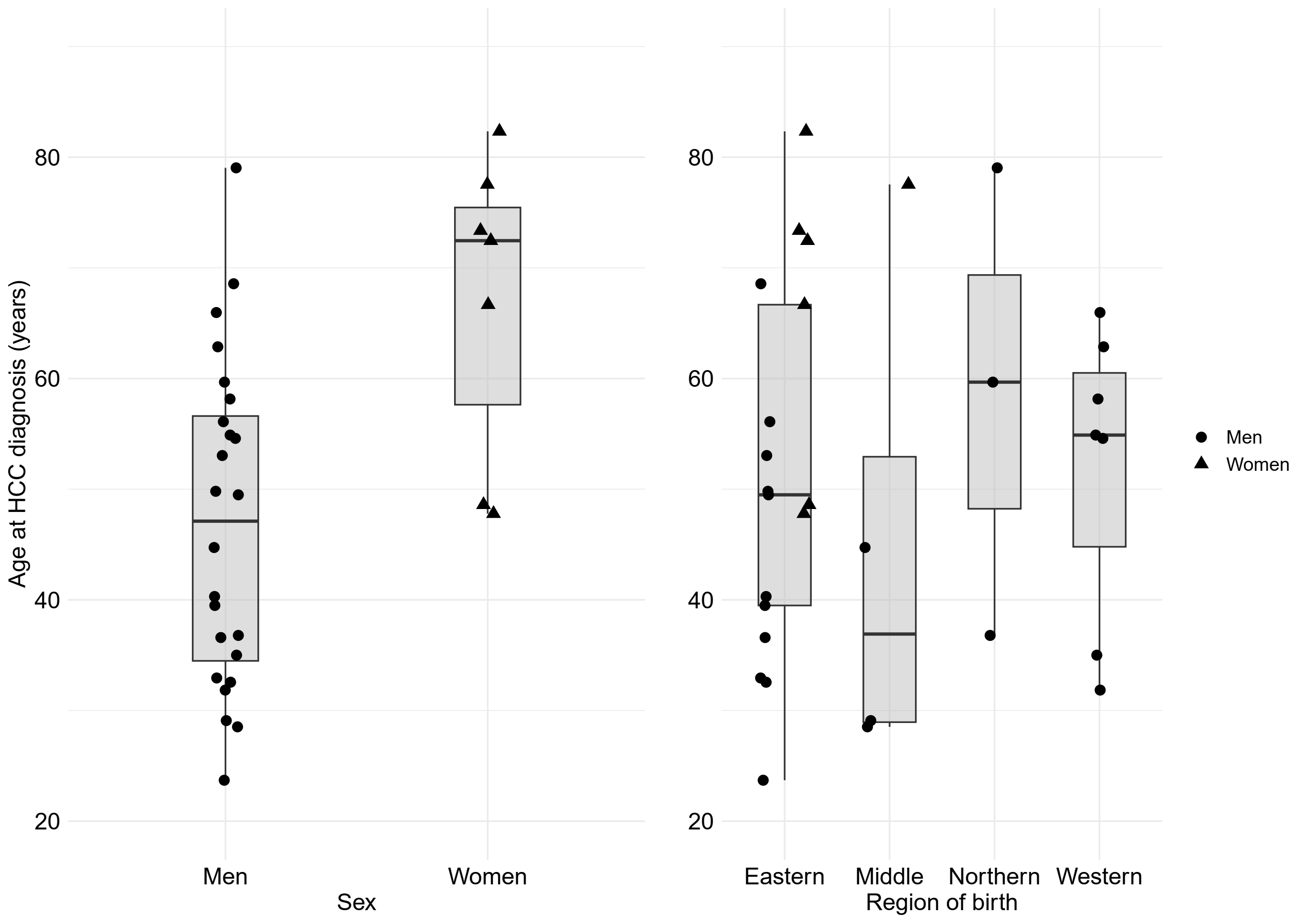


*Dots and triangles represent individual persons with developed HCC(n=31), the middle line (thickened) in the box plot represents the median age at HCC diagnosis. The upper and lower edges of the box represent the 25^th^ and the 75^th^ percentile respectively, and the upper and lower error bars represent the minimum and maximum age, respectively. Y-axis represents age in years at HCC diagnosis. X-axis represent the region of birth and sex. CHB=chronic hepatitis B*

**Figure 3: The incidence rates of hepatocellular carcinoma (HCC) per 100 person-year with 95% confidence intervals in African-born persons with CHB and without cirrhosis at baseline, by sex.**


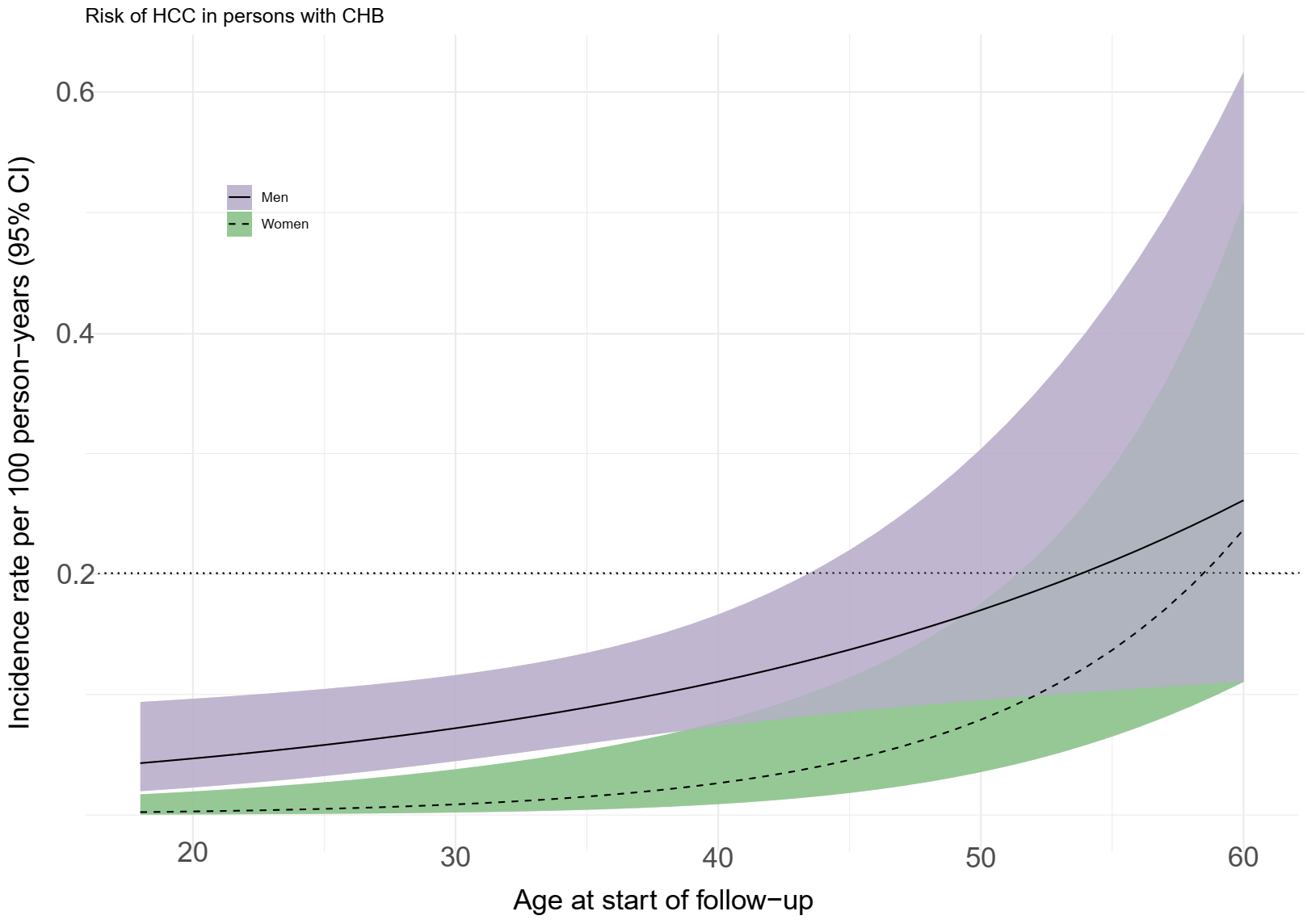


*X-axis represents age at baseline on contiuous scale. Y-axis represents the incidence rate of HCC per 100 persons per year. The dotted horizontal line marks incidence rate 0.2% of cost-effective HCC surveillance in individuals without cirrhosis. The lilac and green zones represent the 95% CI for the mean incidence rate in men and women, respectively. The 0.2% IR was exceeded in men at age 54 years (IR=0.20/100PYs, 95%CI 0.10-0.40) and in women at age 59 years (IR=0.21/100 PYs, 95%CI 0.10-0.45). Age*sex interaction was IRR (0.94, 95% CI 0.88-0.99) suggesting that the increased risk was more pronounced in younger age in men and attenuated with older age compared to women. Abbreviations: HCC= hepatocellular carcinoma; CHB=chronic hepatitis B; CI=confidence interval.*

**Figure S1: The incidence rates of hepatocellular carcinoma (HCC) per 100 person-year with 95% confidence intervals in African-born persons with chronic hepatitis B (CHB) and without cirrhosis, in persons with HCV or HDV co-infection (Figure a), and per African region of birth (Figure b).**

**a**

| 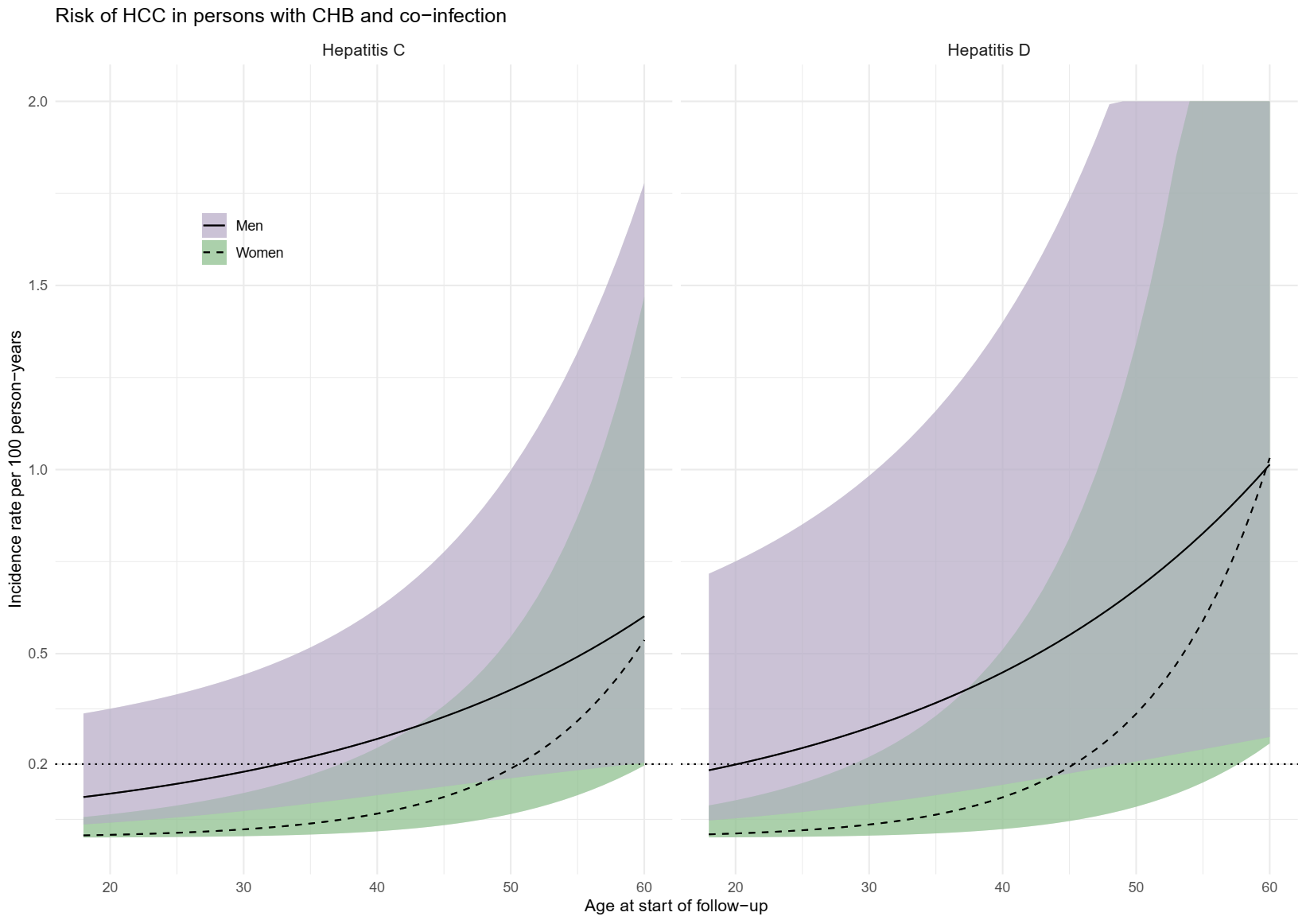  *In persons with HBV/HCV co-infection, the 0.2% surveillance threshod was crossed at age 33 years-old in men (IR=0.20/100PYs, 95% CI 0.08-0.48) and in women at age 51 years-old (IR=0.20/100PYs,95% CI 0.07-0.60). In persons with HBV/HDV co-infection, the threshold was crossed at 20 years-old in men (IR=0.21/100PYs, 95% CI 0.05-0.75) and at 46 years-old in women (IR=0.20/100PYs, 95% CI 0.05-0.90). The lilac and green zones represent the 95% CI for the mean incidence rate in men and women, respectively.* |
| --- |
| 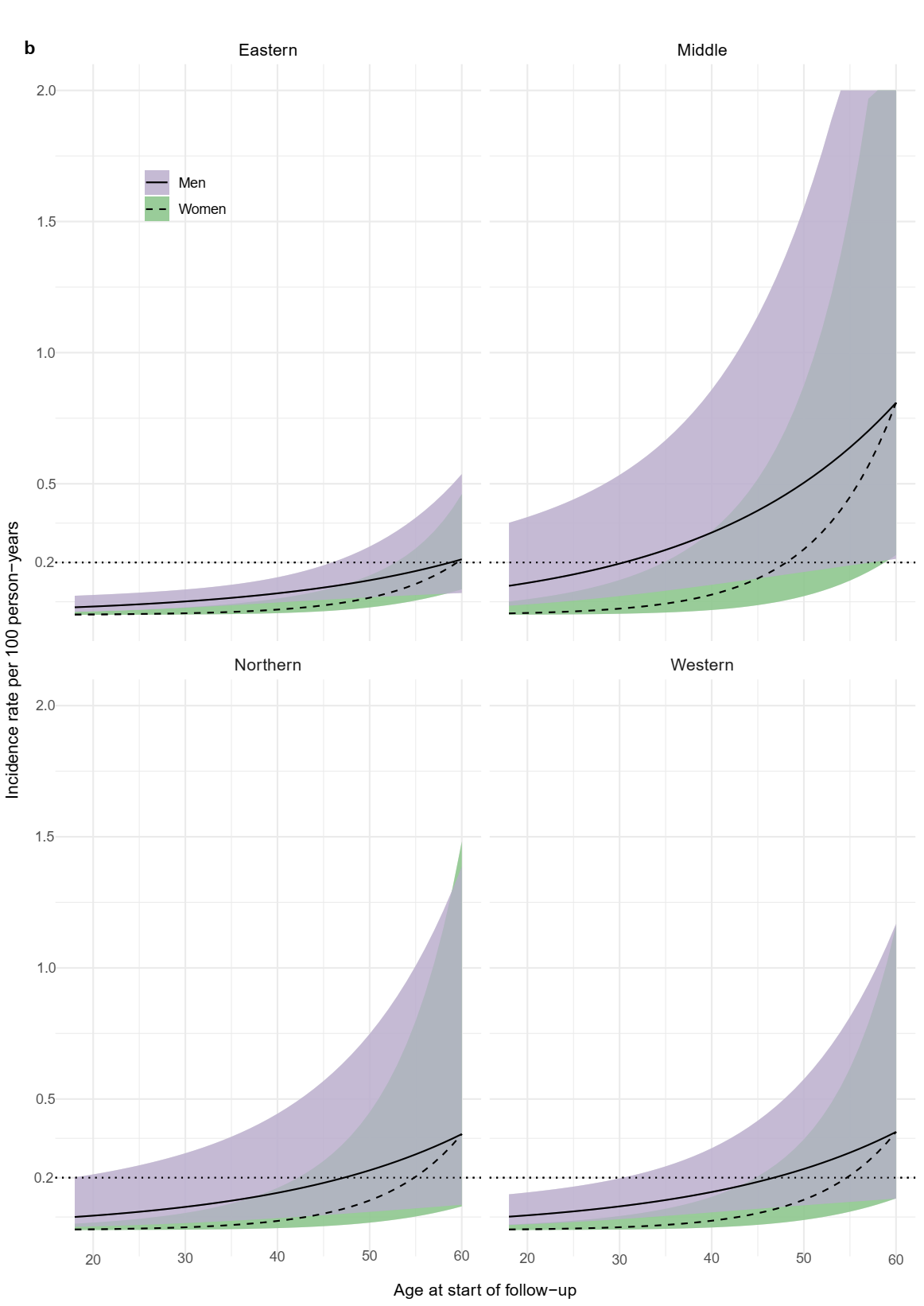 |

*Abbreviations: HCC= hepatocellular carcinoma; CHB=chronic hepatitis B; CI=confidence interval.*

*The lilac and green zones represent the 95% CI for the mean incidence rate in men and women, respectively.*

| **Figure S2: The incidence rate of HCC with 95% confidence intervals in African-born men and women with chronic hepatitis B (CHB), and in matched comparators from the same country of origin and in the general population. Y-axis represents IR/100PYs. X-axis represents the age at baseline.** |
| --- |
| 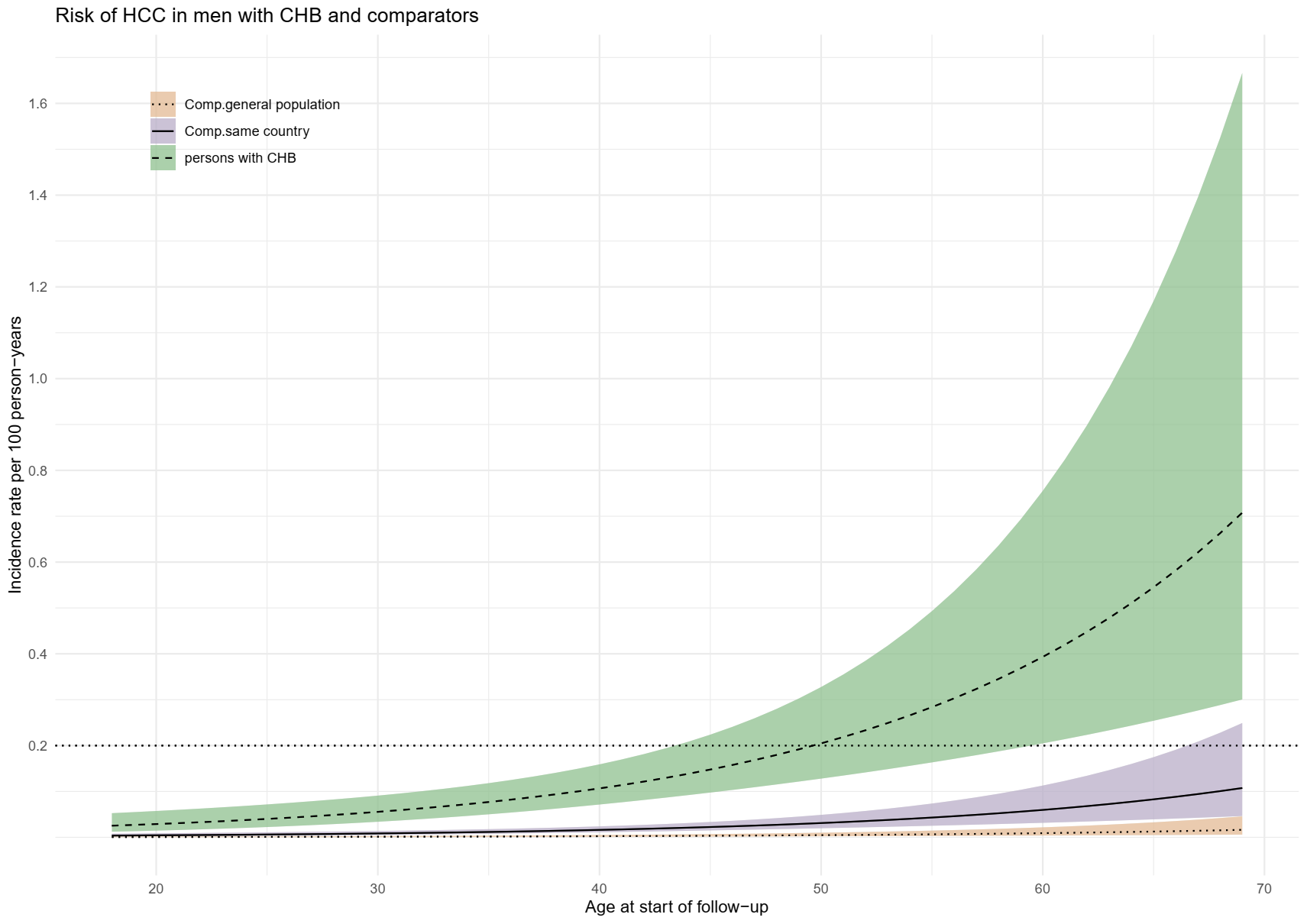  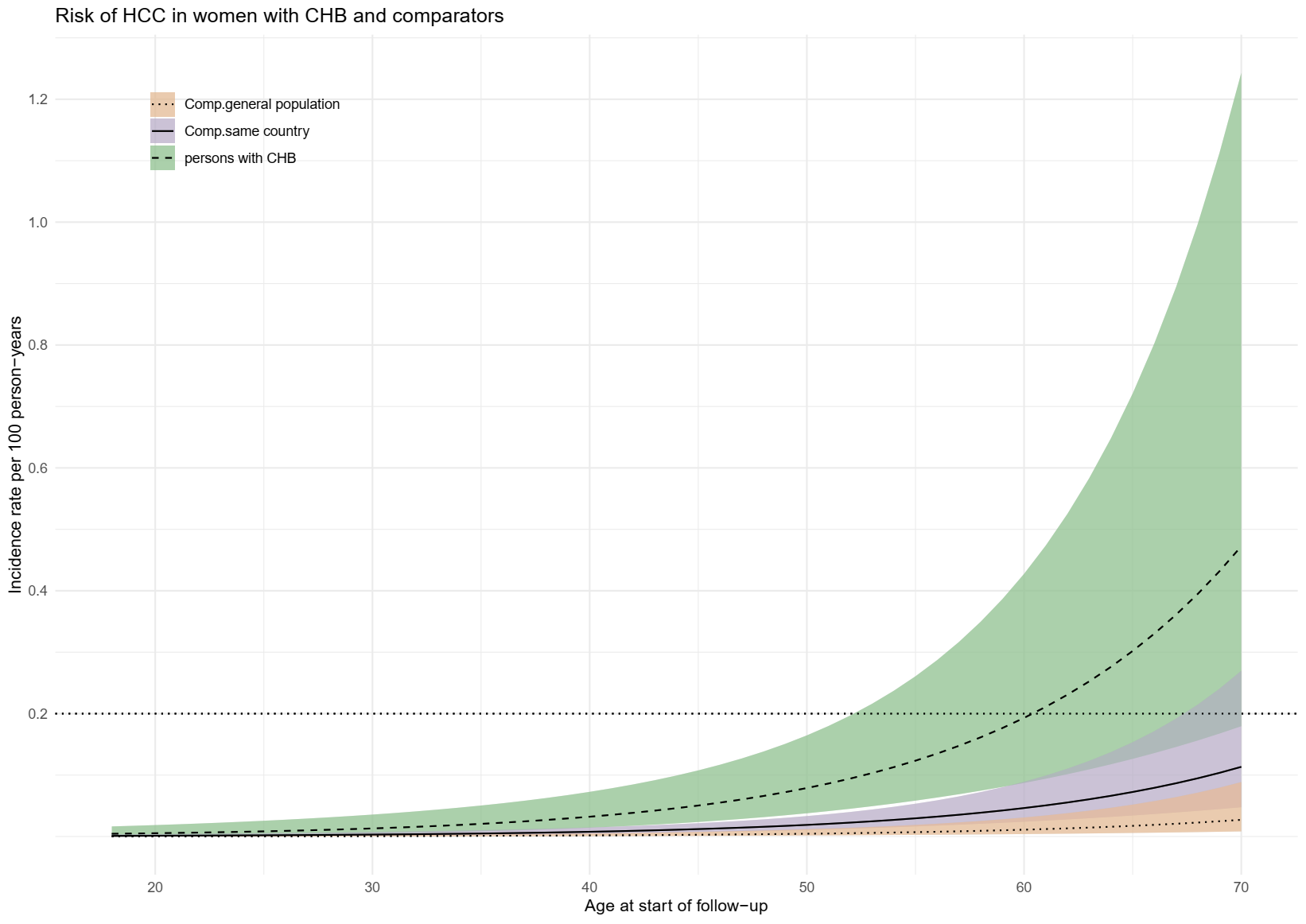  *Abbreviations: CHB=chronic hepatitis B; HCC=hepatocellular carcinoma* |
